# Comparing the mRNA expression profile and the genetic determinism of intramuscular fat traits in the porcine *gluteus medius* and *longissimus dorsi* muscles

**DOI:** 10.1186/s12864-019-5557-9

**Published:** 2019-03-04

**Authors:** Rayner González-Prendes, Raquel Quintanilla, Emilio Mármol-Sánchez, Ramona N. Pena, Maria Ballester, Tainã Figueiredo Cardoso, Arianna Manunza, Joaquim Casellas, Ángela Cánovas, Isabel Díaz, José Luis Noguera, Anna Castelló, Anna Mercadé, Marcel Amills

**Affiliations:** 1 Department of Animal Genetics, Centre for Research in Agricultural Genomics (CRAG), CSIC-IRTA-UAB-UB, Universitat Autònoma de Barcelona, 08193 Bellaterra, Spain; 2 Animal Breeding and Genetics Program, Institute for Research and Technology in Food and Agriculture (IRTA), Rovira Roure 191, 25198 Lleida, Spain; 3 Departament de Ciència Animal, Universitat de Lleida-Agrotecnio Centre, 25198 Lleida, Spain; 4 CAPES Foundation, Ministry of Education of Brazil, Brasilia, DF 70.040-020 Brazil; 5 Departament de Ciència Animal i dels Aliments, Facultat de Veterinària, Universitat Autònoma de Barcelona, 08193 Bellaterra, Spain; 6 Centre for Genetic Improvement of Livestock, Department of Animal Biosciences, University of Guelph, 50 Stone Road East, Guelph, Ontario N1G 2W1 Canada; 7 Institute for Research and Technology in Food and Agriculture (IRTA), Tecnologia dels Aliments, 17121 Monells, Spain

**Keywords:** Intramuscular fat, Fatty acid content, Genome-wide association analysis, Duroc pig, Gene expression

## Abstract

**Background:**

Intramuscular fat (IMF) content and composition have a strong impact on the nutritional and organoleptic properties of porcine meat. The goal of the current work was to compare the patterns of gene expression and the genetic determinism of IMF traits in the porcine *gluteus medius* (GM) and *longissimus dorsi* (LD) muscles.

**Results:**

A comparative analysis of the mRNA expression profiles of the pig GM and LD muscles in 16 Duroc pigs with available microarray mRNA expression measurements revealed the existence of 106 differentially expressed probes (fold-change > 1.5 and q-value < 0.05). Amongst the genes displaying the most significant differential expression, several loci belonging to the Hox transcription factor family were either upregulated (*HOXA9*, *HOXA10*, *HOXB6*, *HOXB7* and *TBX1*) or downregulated (*ARX*) in the GM muscle. Differences in the expression of genes with key roles in carbohydrate and lipid metabolism (e.g. *FABP3*, *ORMDL1* and *SLC37A1*) were also detected. By performing a GWAS for IMF content and composition traits recorded in the LD and GM muscles of 350 Duroc pigs, we identified the existence of one region on SSC14 (110–114 Mb) displaying significant associations with C18:0, C18:1(n-7), saturated and unsaturated fatty acid contents in both GM and LD muscles. Moreover, we detected several genome-wide significant associations that were not consistently found in both muscles. Further studies should be performed to confirm whether these associations are muscle-specific. Finally, the performance of an eQTL scan for 74 genes, located within GM QTL regions and with available microarray measurements of gene expression, made possible to identify 14 *cis*-eQTL regulating the expression of 14 loci, and six of them were confirmed by RNA-Seq.

**Conclusions:**

We have detected significant differences in the mRNA expression patterns of the porcine LD and GM muscles, evidencing that the transcriptomic profile of the skeletal muscle tissue is affected by anatomical, metabolic and functional factors. A highly significant association with IMF composition on SSC14 was replicated in both muscles, highlighting the existence of a common genetic determinism, but we also observed the existence of a few associations whose magnitude and significance varied between LD and GM muscles.

**Electronic supplementary material:**

The online version of this article (10.1186/s12864-019-5557-9) contains supplementary material, which is available to authorized users.

## Background

Intramuscular fat (IMF) content and fatty acids (FA) composition have important effects on the oxidative stability, tenderness and juiciness of pig meat [1]. These phenotypes are moderately heritable and, in consequence, they can be improved through artificial selection [2]. Several genome-wide association studies (GWAS) have been carried out in pigs to identify quantitative trait loci (QTL) influencing IMF content and composition traits [3–12]. Population sizes employed in these studies have oscillated between 138 [12] and 2326 [11] individuals. Indeed, IMF content and composition traits are not routinely recorded by the pig breeding industry despite their strong impact on the manufacturing of cured products because they are difficult and expensive to measure. All these studies have investigated the genomic architecture of IMF traits in a single muscle, so we do not know yet whether the genetic determinism of IMF content and composition is shared across muscles. In a number of GWAS for IMF phenotypes, genome scans for expression QTL (eQTL) have been carried out as a strategy to pinpoint potential causal mutations. Hundreds of eQTL associated with muscle gene expression phenotypes have been identified, and several of them have been shown to co-localize with QTL for traits of economic interest [8, 13–20].

In a previous study, we determined the IMF content and composition of two muscles, *longissimus dorsi* (LD) and *gluteus medius* (GM), in a commercial population of 350 Duroc pigs [21]. Phenotypic correlations between FA traits in these two muscles displayed moderate values ranging from r_P_ = 0.28 to 0.58 [21]. These results suggested the existence of potential differences in the genetic determinism of FA composition across muscles. The goal of the current work was to investigate whether differences exist in the mRNA expression profiles of the GM and LD muscles. Moreover, we aimed to identify QTL for IMF content and composition traits in the GM and LD muscles of Duroc pigs and to establish their positional concordance.

## Results

### Differential mRNA expression in the *gluteus medius* and *longissimus dorsi* muscles

**Fig. 1:**
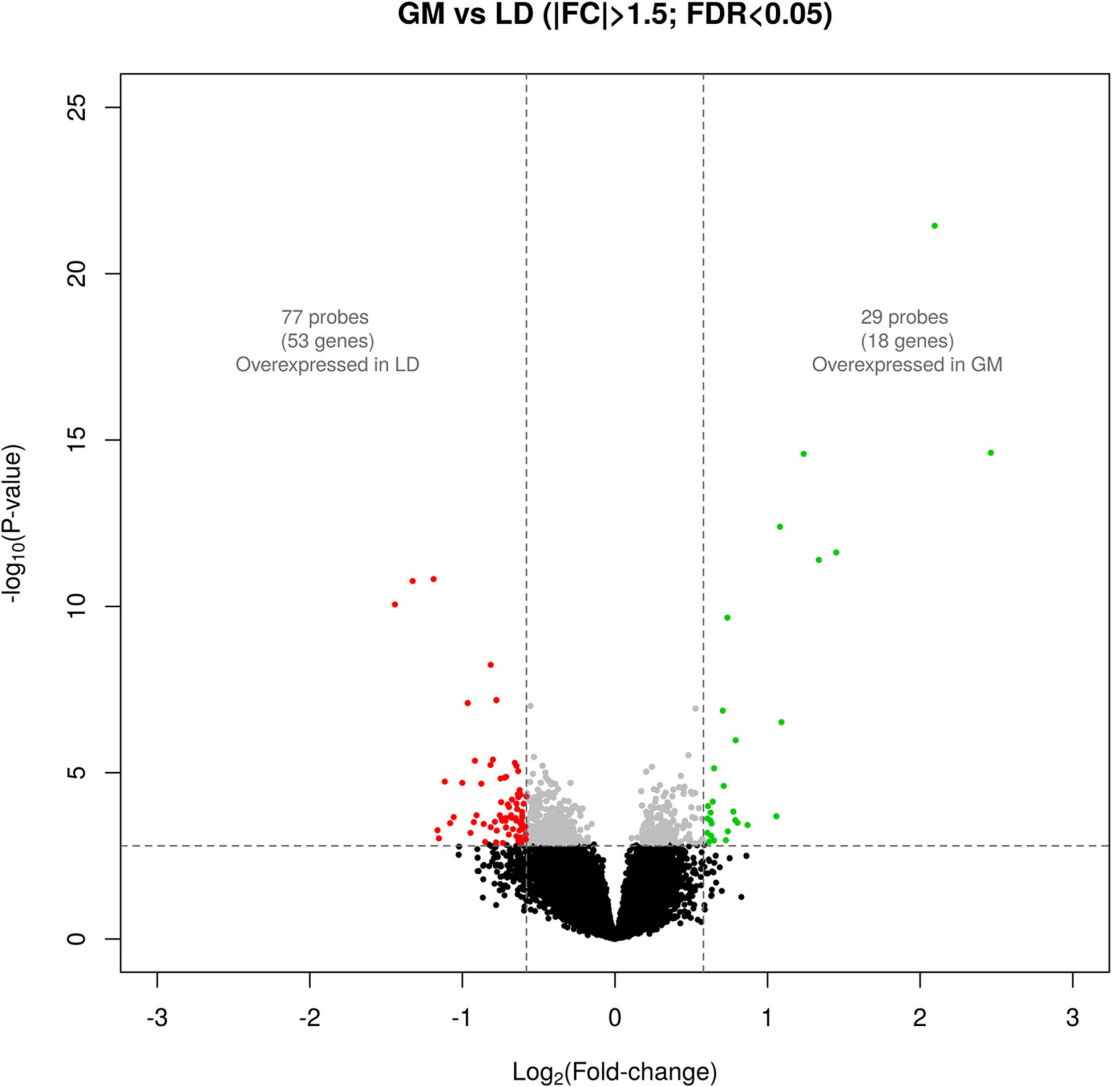
Volcano plot of probes differentially expressed in the *gluteus medius* (GM) and *longissimus dorsi* (LD) muscles, along with fold changes (ratio between GM/LD mean expression values) for these probes (|FC| > 1.5, q-value < 0.05)

As shown in Table 1**,** Fig. 1 and Additional file 1, the comparison of the mRNA expression profiles of the GM and LD muscles based on microarray data highlighted the existence of 106 DE probes (|fold-change(FC)| > 1.5, q-value < 0.05). Amongst the genes displaying the most significant differential expression, several loci belonging to the Hox transcription factor family were either upregulated (*HOXA9, HOXA10, HOXB6, HOXB7* and *TBX1*) or downregulated (*ARX*) in the GM muscle. Differences in the expression of genes with key roles in carbohydrate and lipid metabolism (e.g. *FABP3*, *ORMDL1* and *SLC37A1*) were also detected. Indeed, the pathway analysis revealed that lipid and carbohydrate metabolic processes are enriched in the set of DE genes, although only at the nominal level (Additional file 2).

**Table 1:** List of the twenty genes displaying the highest differential expression between the *gluteus medius* and *longissimus dorsi* muscles (threshold of significance: |FC| > 1.5, q-value < 0.05) ^1^

Microarray probe	Gene	Fold Change	-log_10_(*P*)	-log_10_(*q*)
Ssc.27606.1.S1_at	*HOXA10*	2.36	14.58	10.68
Ssc.20706.1.S1_at	*HOXB6*	2.73	11.62	7.94
Ssc.14356.1.A1_at	*LAMA2*	−2.28	10.82	7.29
Ssc.2743.1.S1_at	*MSS51*	−2.72	10.06	6.63
Ssc.22336.2.A1_at	*HOXB7*	1.67	9.66	6.28
Ssc.1294.1.S1_at	*NIT1*	−1.71	7.18	3.87
Ssc.17238.1.A1_at	*ITIH4*	−1.95	7.09	3.82
Ssc.4214.1.A1_at	*SLC37A1*	1.63	6.87	3.69
Ssc.26748.1.A1_at	*HOXA9*	2.13	6.52	3.37
Ssc.28628.1.S1_at	*TIAL1*	−1.58	5.30	2.28
Ssc.14245.1.A1_at	*ZFP92*	−1.56	5.20	2.24
Ssc.8360.1.A1_at	*INPP5F*	−1.76	5.23	2.24
Ssc.21802.1.S1_at	*CCL19*	1.57	5.13	2.20
Ssc.3838.1.S1_at	*COA3*	−1.55	5.05	2.13
Ssc.22770.1.A1_at	*ARX*	−1.64	4.88	2.03
Ssc.4076.1.S1_at	*PRUNE2*	−1.65	4.85	2.02
Ssc.1664.1.A1_at	*NRN1*	−2.17	4.73	1.95
Ssc.9291.1.A1_at	*PALMD*	−1.84	4.67	1.94
Ssc.4360.1.A1_at	*FABP3*	1.64	4.60	1.91
Ssc.11423.2.A1_at	*ORMDL1*	1.54	4.48	1.85

^1^Fold-change refers to mean expression levels in *gluteus medius* compared to *longissimus dorsi*; −log_10_(*P*): decimal logarithm of the nominal *P*-value, −log_10_(*q*): decimal logarithm of the *q*-value

### Genome-wide association study of intramuscular fat phenotypes

**Fig. 2:**
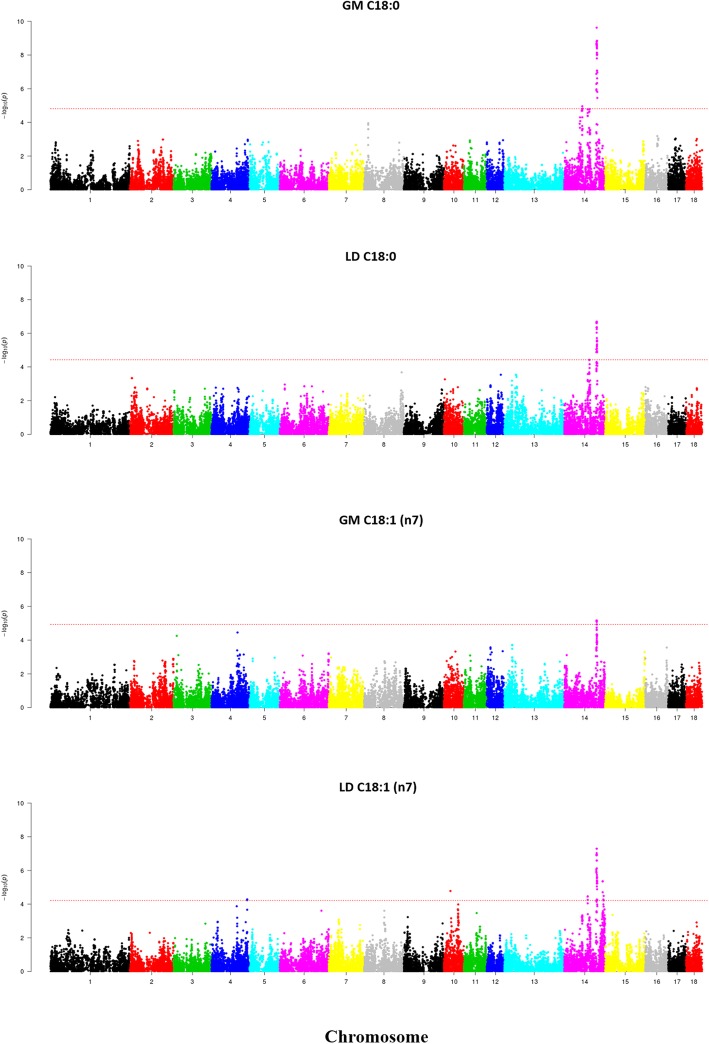
Manhattan plots depicting the genome-wide significant associations between SNP markers and C18:0 and C18:1(n-7) fatty acid contents in the *gluteus medius* (GM) and *longissimus dors*i (LD) muscles of Duroc pigs. Negative log_10_ *P*-values of the associations between SNPs and phenotypes are plotted against the genomic location of each marker SNP. Markers on different chromosomes are denoted by different colors. The dashed line represents the genome-wide threshold of significance (q-value ≤0.05). It can be seen a strong association between SNPs on SSC14 (110–114 Mb) and C18:0 and C18:1 (n-7) recorded in both muscles (GM and LD)

**Fig. 3:**
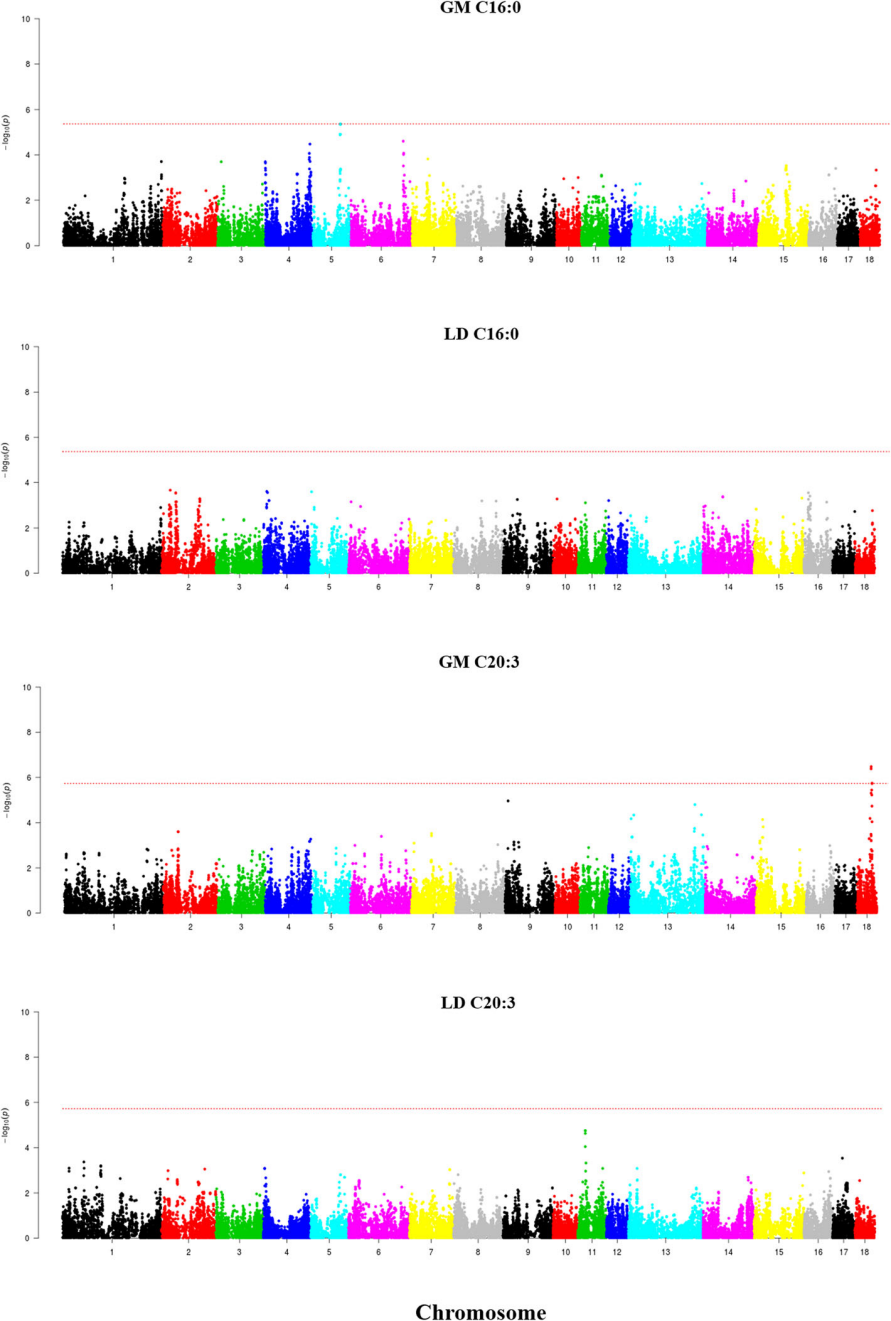
Manhattan plots depicting the genome-wide significant associations between SNP markers and C16:0 and C20:3 fatty acid contents in the *gluteus medius* (GM) and *longissimus dors*i (LD) muscles of Duroc pigs. Negative log_10_ *P*-values of the associations between SNPs and phenotypes are plotted against the genomic location of each SNP. Markers on different chromosomes are denoted by different colors. The dashed line represents the genome-wide threshold of significance (q-value ≤0.05). It can be seen that SNPs at SSC5 and SSC18 are associated, respectively, with C16:0 and C20:3 recorded in the GM muscle but not in the LD one

**Fig. 4:**
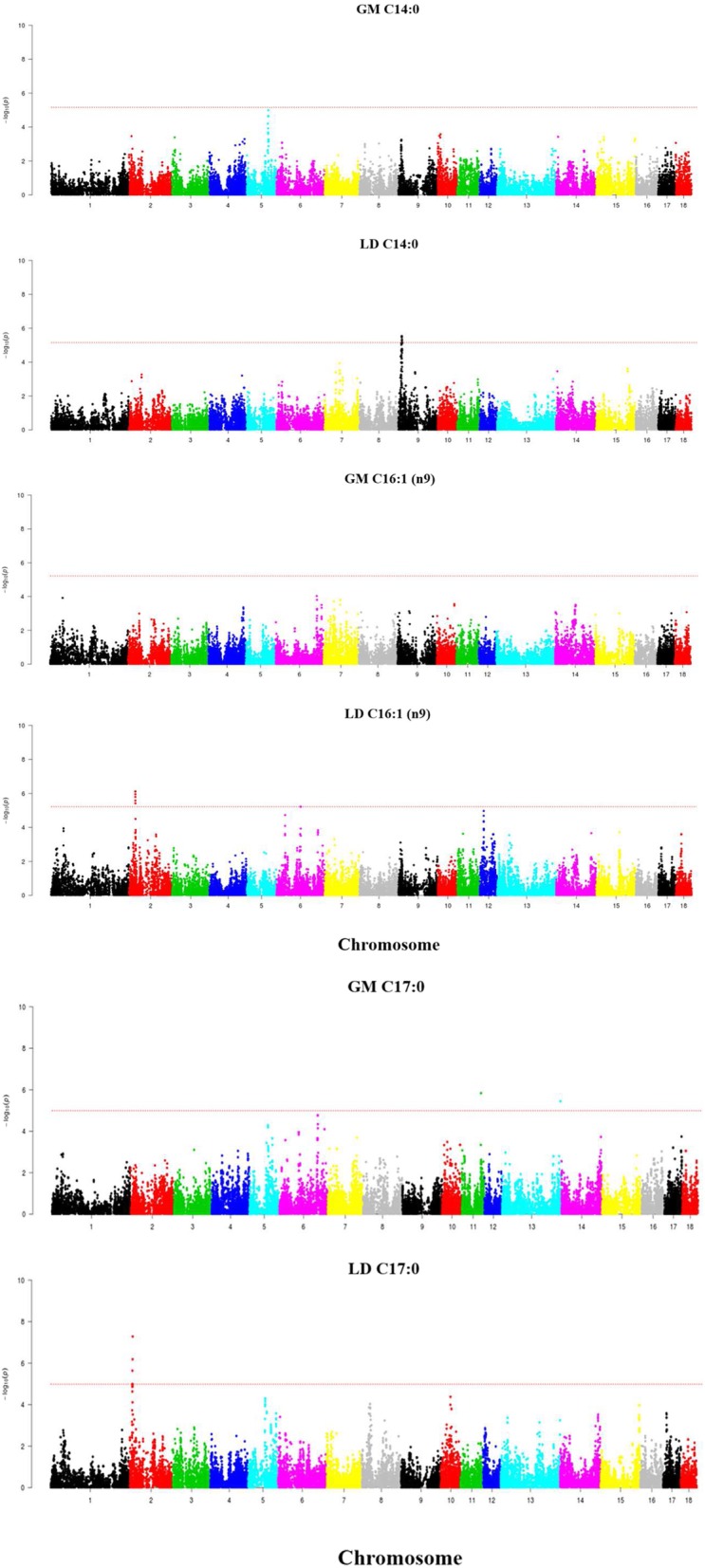
Manhattan plots depicting the genome-wide significant associations between SNPs and C14:0, C16:1(n-9) and C17:0 fatty acid contents in the *gluteus medius* (GM) and *longissimus dors*i (LD) muscles of Duroc pigs. Negative log_10_ *P*-values of the associations between SNPs and phenotypes are plotted against the genomic location of each SNP. Markers on different chromosomes are denoted by different colors. The dashed line represents the genome-wide threshold of significance (q-value ≤0.05). It can be seen that SNPs at SSC2 (C16:1 and C17:0) and SSC9 (C14:0) display significant associations with FA composition traits recorded in the LD muscle but not in the GM one

Performance of a GWAS revealed the existence of several QTL displaying genome-wide significant associations with IMF phenotypes (Table 2 and Figs. 2, 3 and 4). With regard to IMF content, we found a region on SSC6 (146.5–147.7 Mb) which showed a genome-wide significant association in the LD (−log_10_*P*-value = 6.88, q-value = 0.0044, δ **=** − 0.69 ± 0.13) and GM (−log_10_*P*-value = 6.47, q-value = 0.01, δ **=** − 0.84 ± 0.16) muscles only when backfat was removed as a covariate in the statistical analysis. When considering IMF composition, the largest number of associations were found in SSC14, where we observed the existence of one region located between 110 and 114 Mb and displaying significant associations with C18:0, C18:1 (n-7), saturated and unsaturated FA contents both in the GM and the LD muscles (Fig. 2). Effect sizes of markers were negative for C18:0 (δ_GM_ = − 0.63 ± 0.10, δ_LD_ = − 0.62 ± 0.10) and saturated FA - SFA (δ_GM_ = − 0.87 ± 0.18, δ_LD_ = − 0.95 ± 0.19) and positive for C18:1 (n-7) (δ_GM_ = 0.13 ± 0.03, δ_LD_ = 0.18 ± 0.03) and unsaturated FA - UFA (δ_GM_ = 0.87 ± 0.18, δ_LD_ = 0.94 ± 0.19). We also observed significant associations between GM C18:0 and three additional SSC14 regions on 54.6–55.3 Mb, 60.6–62.9 Mb and 81.1–87.7 Mb. This latter region was also associated with LD SFA. Regarding C18:1(n-7) FA content in the LD muscle, two regions on SSC14 at 81.2–81.3 Mb and 133.1–136.7 Mb were associated with this trait (Table 2, Fig. 2). Moreover, we identified several regions showing associations that were not consistently found in both muscles (Table 2 and Figs. 3 and 4). Two QTL for C16:0 (SSC5: 76.2–77.1 Mb) and C20:3 (n-3) (SSC18: 39.4–42.3 Mb) were significant for the GM muscle but not for the LD one (Fig. 3). Moreover, the effect size of the QTL for C16:0 on SSC5 (76.2–77.1 Mb) was considerably high (δ_GM_ = − 0.87 ± 0.20). Conversely, three QTL for C14:0 (SSC9: 10.4–13.2 Mb), C16:1 (n-9) (SSC2: 21.9–22.3 Mb) and C17:0 (SSC2: 10.6–11.7 Mb) were significant for the LD but not for the GM muscle (Fig. 4**,** Table 2). However, the effects sizes of these three QTL were close to zero, so it is difficult to evaluate their relevance. A GWAS signal was observed for GM C14:0 on SSC9:10–13 Mb, but it did not reach genome-wide significance (Fig. 4).

**Table 2:** Genome-wide significant QTL for intramuscular fat composition traits recorded in the *gluteus medius* (GM) and *longissimus dorsi* (LD) muscles of Duroc pigs^1^

Main FA (g*luteus medius*)										
Traits	SSC	N	SNP	Region (Mb)	-log_10_(*P*)	*q*-value	B	δ ± SE	A_1_	MAF
GM C16:0	5	9	H3GA0016883	76.2–77.1	4.89	0.047	0.42	- 0.87 ± 0.20	A	0.07
GM C18:0	14	2	ASGA0063465	54.6–55.3	4.30	0.027	1	- 0.44 ± 0.11	G	0.23
		9	ALGA0078300	60.6–62.9	4.96	0.008	0.36	- 0.48 ± 0.11	C	0.23
		8	ALGA0079209	81.1–87.7	4.77	0.011	0.55	0.43 ± 0.09	G	0.48
		42	ALGA0081091	110.9–114.5	9.20	0.000	0.00	- 0.63 ± 0.10	A	0.35
GM C18:1 (n-7)	14	14	ALGA0081091	111.4–112.7	5.15	0.024	0.23	0.13 ± 0.03	A	0.35
GM SFA	14	37	ALGA0081091	111.4–113.8	5.51	0.007	0.10	- 0.87 ± 0.18	A	0.35
GM UFA	14	37	ALGA0081091	111.4–113.8	5.52	0.007	0.10	0.87 ± 0.18	A	0.35
Main FA (*longissimus dorsi*)										
Traits	SSC	N	SNP	Region (Mb)	-log_10_(*P*)	*q*-value	B	δ ± SE	A_1_	MAF
LD C18:0	14	2	CASI0010207	86.8–86.8	4.43	0.035	1	0.47 ± 0.11	G	0.41
		36	ALGA0081091	110.9–113.6	6.62	0.001	0.01	- 0.62 ± 0.10	A	0.35
LD C18:1 (n-7)	14	4	ALGA0079221	81.2–81.3	4.45	0.025	1	- 0.13 ± 0.03	G	0.45
		43	ALGA0081091	110.9–114.4	6.92	0.000	0.00	0.18 ± 0.03	A	0.35
		6	ALGA0082693	133.1–136.7	5.36	0.005	0.14	- 0.13 ± 0.03	A	0.46
LD SFA	14	6	ASGA0064951	86.4–87.7	4.57	0.028	0.88	0.81 ± 0.17	A	0.41
		33	ALGA0081091	110.9–113.6	5.92	0.004	0.04	- 0.95 ± 0.19	A	0.35
LD UFA	14	31	ALGA0081091	110.9–113.6	5.81	0.004	0.05	0.94 ± 0.19	A	0.35
Minor FA (*gluteus medius*)										
Traits	SSC	N	SNP	Region (Mb)	-log_10_(*P*)	*q*-value	B	δ ± SE	A_1_	MAF
GM C20:3 (n-3)	18	8	ASGA0097792	39.4–42.3	6.38	0.005	0.01	0.12 ± 0.02	A	0.07
Minor FA (*longissimus dorsi*)										
Traits	SSC	N	SNP	Region (Mb)	-log_10_(*P*)	*q*-value	B	δ ± SE	A_1_	MAF
LD C14:0	9	33	M1GA0026515	10.4–13.2	5.11	0.014	0.25	0.09 ± 0.02	A	0.41
LD C16:1 (n-9)	2	6	H3GA0006292	21.9–22.3	6.11	0.012	0.03	- 0.02 ± 0.00	A	0.25
LD C17:0	2	10	MARC0050503	10.6–11.7	7.30	0.002	0.00	- 0.03 ± 0.01	A	0.30

^1^SSC: porcine chromosome, N: Number of SNPs significantly associated with the trait under study, SNP: SNP displaying the most significant association with the trait under study, Region (Mb): region containing SNPs significantly associated with the trait under study, −log_10_ (*P*): decimal logarithm of the nominal *P*-value, *q*-value: *q*-value calculated with a false discovery rate approach, *B*: Bonferroni corrected *P*-values, δ: effect size of the marker and its standard error (SE), A_1_: minor allele, MAF: minor allele frequency

This apparent lack of concordance between the associations found in GM and LD might be due to technical reasons. For instance, the high stringency of the correction for multiple testing could increase the rate of false negatives. Limited statistical power may also lead to the occurrence of a high type 2 error rate, so we have estimated the power of our GWAS by using the procedure reported by Purcell et al. [22]. With a sample size of 350 pigs, we should be able to detect alleles with moderate to large effects (Additional file 3), while the majority of alleles with small effects would be missed. To confirm part of the associations found, we genotyped 12 SNPs mapping to six protein-coding genes i.e. *SLC38A1* (SSC5: 77.0–77.0 Mb), *SLC38A4* (SSC5: 77.4–77.4 Mb), *UVRAG* (SSC9: 10.0–10.3 Mb), *KCNIP2* (SSC14: 112.8–112.8 Mb), *BLOC1S2* (SSC14: 111.3–111.3 Mb) and *SCD* (SSC14: 111.4–111.4 Mb) loci (Table 3). Several microRNA and lincRNA genes map to QTL regions (Additional file 4), but their functions are mostly unknown so it is difficult to make biological inferences from these co-localizations.

**Table 3:** Association analyses between 12 polymorphisms in six candidate genes and intramuscular fat content and composition traits measured in the *gluteus medius* (GM) and *longissimus dorsi* (LD) muscles of Duroc pigs^1^

Gene	SNP	Type	Trait	q-value	δ (SE)	A_1_	MAF
SSC5 (76.2–77.1 Mb)							
*SLC38A1*	rs341329842 (5:77.02 Mb)	Splice region (T/C)	GM C16:0	0.000	−0.61 (0.15)	C	0.15
			LD C16:0	0.161	−0.27 (0.15)		
*SLC38A4*	rs333018168 (5:77.44 Mb)	Missense (G/C, Q24E)	GM C16:0	0.014	−0.51 (0.19)	C	0.13
			LD C16:0	0.266	−0.22 (0.19)		
SSC9 (10.4–13.2 Mb)							
*UVRAG*	rs321243508 (9:10.31 Mb)	Missense (A/C, K286Q)	GM C14:0	0.005	0.07 (0.02)	C	0.43
			LD C14:0	0.001	0.08 (0.02)		
	rs328455999 (9:10.33 Mb)	Missense (G/A, R303H)	GM C14:0	0.005	0.05 (0.02)	G	0.26
			LD C14:0	0.001	0.07 (0.02)		
SSC14 (110–114 Mb)							
*BLOC1S2*	rs335981556 (14:111.39 Mb)	Missense (G/A, L6F)	GM C18:0	0.001	−0.62 (0.13)	A	0.37
			LD C18:0	0.003	−0.50 (0.10)		
			GM SFA	0.077	−0.71 (0.32)		
			LD SFA	0.030	−0.68 (0.26)		
			GM UFA	0.008	0.71 (0.32)		
			LD UFA	0.032	0.67 (0.26)		
*KCNIP2*	rs320607389 (14:112.86 Mb)	Missense (G/A, A50V)	GM C18:0	0.001	−0.58 (0.14)	G	0.38
			LD C18:0	0.003	−0.51 (0.10)		
*SCD*	rs698797651 (14:111.4608 Mb)	Upstream (G/GC)	GM C18:0	0.001	−0.59 (0.14)	G	0.36
			LD C18:0	0.005	−0.49 (0.10)		
	rs323081995 (14:111.4616 Mb)	5′-UTR (T/C)	GM C18:0	0.001	−0.63 (0.13)	C	0.37
			LD C18:0	0.003	−0.51 (0.10)		
			GM SFA	0.049	−0.80 (0.32)		
			LD SFA	0.029	−0.72 (0.27)		
			GM UFA	0.049	0.80 (0.32)		
			LD UFA	0.031	0.71 (0.27)		
	rs80912566 (14:111.4617 Mb)	5′-UTR (C/T)	GM C18:0	0.001	−0.61 (0.13)	T	0.37
			LD C18:0	0.003	−0.51 (0.10)		
			GM SFA	0.049	−0.83 (0.32)		
			LD SFA	0.029	−0.70 (0.27)		
			GM UFA	0.049	0.83 (0.32)		
			LD UFA	0.031	0.69 (0.26)		
	rs342182479 (14:111.4618 Mb)	5′-UTR (G/A)	GM C18:0	0.001	−0.62 (0.13)	A	0.37
			LD C18:0	0.003	−0.51 (0.10)		
			GM SFA	0.049	−0.84 (0.32)		
			LD SFA	0.029	−0.72 (0.27)		
			GM UFA	0.049	0.84 (0.32)		
			LD UFA	0.031	0.71 (0.26)		
	rs45434498 (14:111.473 Mb)	Splice region (G/A)	GM C18:0	0.001	−0.61 (0.13)	A	0.37
			LD C18:0	0.003	−0.52 (0.10)		
			GM SFA	0.049	−0.84 (0.31)		
			LD SFA	0.029	−0.75 (0.27)		
			GM UFA	0.049	0.84 (0.32)		
			LD UFA	0.030	0.74 (0.26)		
	rs713641545 (14:111.474 Mb)	3′-UTR (A/G)	GM C18:0	0.001	−0.63 (0.13)	G	0.37
			LD C18:0	0.003	−0.51 (0.10)		
			GM SFA	0.049	−0.81 (0.32)		
			LD SFA	0.029	−0.73 (0.27)		
			GM UFA	0.049	0.81 (0.32)		
			LD UFA	0.031	0.72 (0.26)		

^1^SSC: porcine chromosome, *q*-value: *q*-value calculated with a false discovery rate approach, **δ**: effect size of the marker and its standard error (SE), A_1_: minor allele, MAF: minor allele frequency

Paralleling the results in the GWAS analysis, the two SNPs in the *SLC38A1* and *SLC38A4* genes showed significant associations with C16:0 in the GM muscle but not in LD (Table 3). Regarding the two SNPs in the *UVRAG* gene, they showed significant associations with C14:0 in both muscles (Table 3). Finally, polymorphisms in the *KCNIP2*, *BLOC1S2* and *SCD* genes displayed significant associations with C18:0 (q-value < 0.005) and SFA (q-value < 0.05) in both muscles, a result that, once again, confirms part of the associations detected in the GWAS (Table 3). The polymorphism of the *SCD* and *BLOC1S2* genes was also associated with UFA content in the GM and LD muscles (Table 3).

We have also investigated whether genes located within QTL regions are DE when comparing the mRNA expression profiles of the LD and GM muscles. If a threshold based on |FC| > 1.5 and q-value < 0.05 is considered, the only locus showing differential expression is the 5′-nucleotidase, cytosolic II (*NT5C2*) gene, which maps to SSC14 (114 Mb). If we consider a lowered significance threshold (|FC| > 1.2 and q-value = 0.05), then six additional genes display DE between GM and LD (Additional file 5).

### Detection of expression QTL for genes located within QTL regions in the *gluteus medius* muscle

**Fig. 5:**
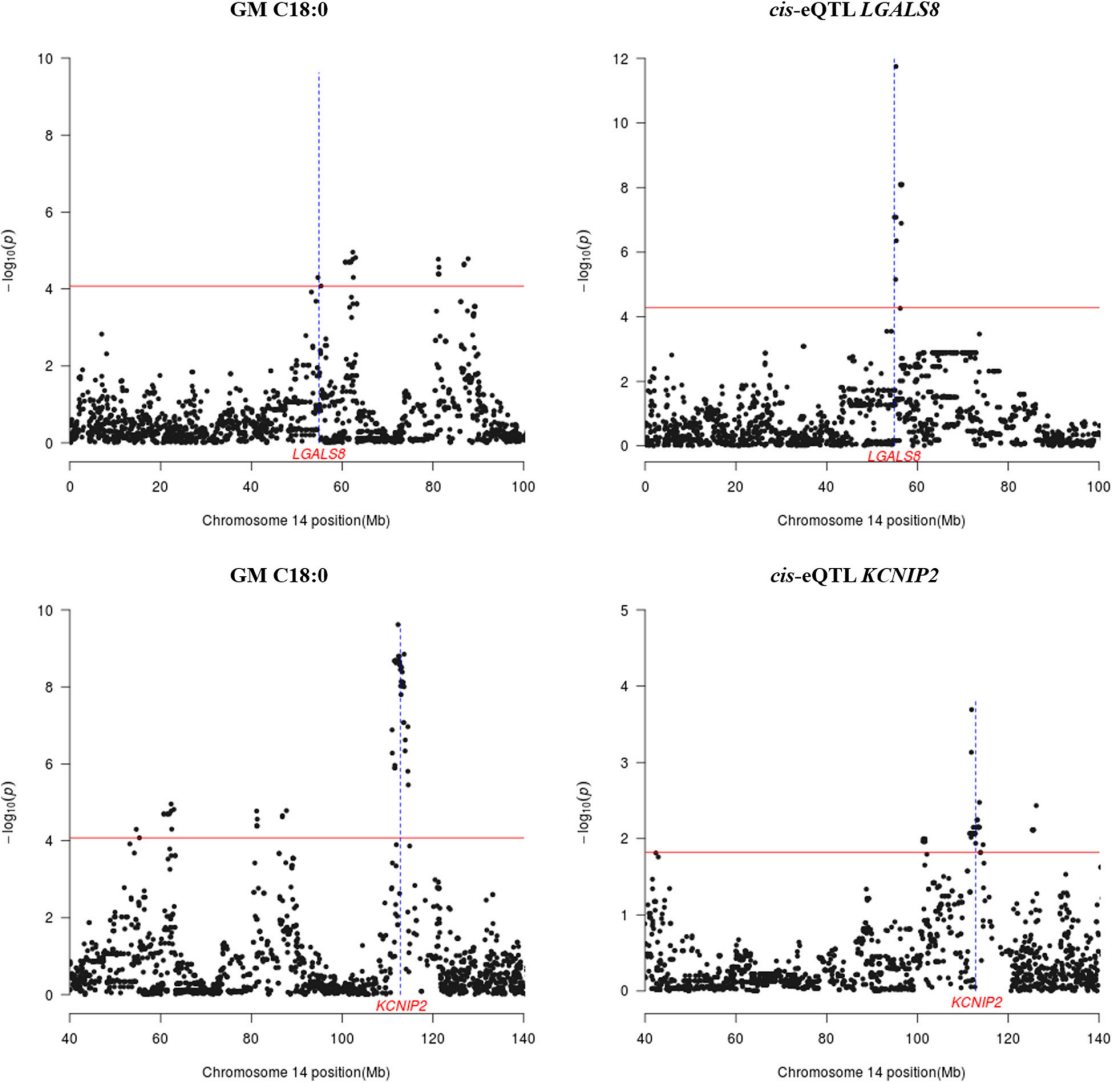
Co-localization of *cis*-eQTL (right panel) for the **a** *LGALS8* and **b** *KCNIP2* genes and two QTL regions (left panel) for *gluteus medius* (GM) C18:0 traits: **a** SSC14, 54.6–55.3 Mb, **b** SSC14, 110–114 Mb. The x-axis represents the chromosomal region (Mb) containing the co-localizing QTL and eQTL, and the y-axis shows the –log_10_ (*P*-value) of the reported associations. The horizontal line indicates the threshold of significance (q-value ≤0.05). The vertical line depicts the genomic location of the *LGALS8* and *KCNIP2* genes

Performance of an eQTL scan for 74 genes, located within the GM QTL regions (as defined in Table 2) and with available microarray measurements of gene expression, made possible to identify 14 *cis*-eQTL regulating the expression of 14 loci (Table 4, Fig. 5). As shown in Table 4, chromosome 14 encompassed most of these *cis*-eQTL, which regulated genes co-localizing with two GM C18:0 QTL at 54.6–55.3 Mb (galectin 8, *LGALS8* and lysosomal trafficking regulator, *LYST*) and 81.1–87.7 Mb (annexin A8 gene, *ANXA8*). The remaining *cis*-regulated genes, i.e. *BLOC1S2*, cytochrome C oxidase assembly homolog (*COX15*), hypoxia inducible factor 1 subunit α inhibitor (*HIF1AN*), F-Box and WD repeat domain containing 4 (*FBXW4*), *KCNIP2* and armadillo-like helical domain containing 3 (*ARMH3*) co-localized with the SSC14 (111.4–113.8 Mb) region displaying pleiotropic effects on muscle FA composition. As shown in Table 4, we have also identified *cis*-regulated genes mapping to a GM C20:3 (n-3) QTL on SSC18 (39.4–42.3 Mb) i.e. FK506 binding protein 14, (*FKBP14*); ɣ-glutamylcyclotransferase (*GGCT*) and WAS/WASL interacting protein family member 3 (*WIPF3*).

**Table 4:** List of the co-localizations between QTL for IMF traits recorded in the *gluteus medius* (GM) muscle and *cis*-eQTL regulating the mRNA levels of genes expressed in the GM muscle and mapping to QTL regions^1^

SSC	N	SNPs	Region (Mb)	-log_10_ (*P*)	*q*-value	B	δ ± SE	A_1_	MAF	Gene Symbol	Region (Mb)	Traits	Region(Mb)
14	7	ASGA0063513	54.2–55.3	12.62	0.000	0.00	0.68 ± 0.08	A	0.37	*LGALS8*	54.8	C18:0	54.6–55.3
	3	H3GA0040331	54.9–56.2	3.05	0.039	0.04	−0.25 ± 0.07	G	0.44	*LYST*	55.4–55.6		
	21	MARC0041893	86.1–88.0	7.96	0.000	0.00	0.82 ± 0.13	A	0.32	*ANXA8*	88.3		81.1–87.7
	1	DBMA0000150	114.0–114.0	2.63	0.023	0.02	0.32 ± 0.10	G	0.45	*TAF5*	114.3	C18:0; C18:1 (n-7); SFA; UFA	110.9–114.5
	26	MARC0043866	110.5–111.9	6.10	0.000	0.00	0.42 ± 0.07	A	0.49	*COX15*	110.8		
	6	DRGA0014486	111.7–112.6	4.23	0.001	0.00	0.33 ± 0.08	G	0.16	*BLOC1S2*	111.3		
	42	ALGA0081091	110.9–113.8	3.03	0.004	0.02	−0.29 ± 0.08	A	0.33	*HIF1AN*	111.6		
	37	ALGA0081091	111.4–113.8	1.98	0.035	0.24	−0.19 ± 0.08	A	0.33	*FBXW4*	112.6–112.7		
	28	MARC0015087	111.4–113.8	3.69	0.005	0.00	0.39 ± 0.10	A	0.17	*KCNIP2*	112.8		
	28	ALGA0081091	110.9–113.8	3.12	0.002	0.02	−0.27 ± 0.08	A	0.33	*ARMH3*	112.8–113.0		
18	9	ASGA0079732	40.0–43.3	2.32	0.023	0.16	0.23 ± 0.08	G	0.23	*AQP1*	42.0	GM C20:3 (n-3)	39.4–42.3
	32	DIAS0000749	41.5–43.9	2.25	0.049	0.18	−0.32 ± 0.10	A	0.35	*GGCT*	42.4		
	21	ALGA0116124	42.0–43.3	3.29	0.009	0.02	0.29 ± 0.08	G	0.35	*FKBP14*	42.9		
	23	ALGA0116124	42.0–43.3	3.27	0.004	0.03	−0.27 ± 0.08	G	0.35	*WIPF3*	42.9–43.0		

^1^SSC: porcine chromosome, N: number of SNPs significantly associated with the trait under study, SNP: SNP displaying the most significant association with the trait under study, Region (Mb): region containing SNPs significantly associated with the trait under study, −log_10_(*P*): decimal logarithm of the nominal *P*-value, *q*-value: *q*-value calculated with a false discovery rate approach, B: Bonferroni-corrected *P*-value, **δ**: effect size of the marker and its standard error (SE), A_1_: minor allele, MAF: minor allele frequency.

With the aim of confirming the existence of these *cis*-eQTL, we have used a previously published RNA-Seq data set comprising 52 of the 103 pigs analysed with microarrays [23]. As shown in Additional file 3, six *cis*-eQTL regulating the expression of six genes (*ANXA8, BLOC1S2, COX15, GGCT, LGALS8* and *FBXW4*) showed significant differences across at least 2 genotypes when mRNA expression means measured with both microarrays and RNA-Seq were compared with a Student’s t-test. Moreover, both microarray and RNA-Seq methods were consistent with regard to the direction of the changes observed when comparing genotypes (Additional file 3).

The absence of an eQTL regulating *SCD* mRNA expression was unexpected because there are evidences that the rs80912566 (g.2228T > C) SNP is associated with the expression of this gene [24]. In Additional file 3, we have compared the GM *SCD* mRNA levels, measured with microarrays in TT, TC and CC pigs. Although TC individuals display a higher *SCD* mRNA expression than the CC and TT ones, differences are not statistically significant. A similar pattern was found when *SCD* mRNA expression was measured by RNA-seq (Additional file 3). We also analysed the correlation between the expression of the genes regulated by *cis*-eQTL and the phenotypes determined by the QTL to which they map to (Additional file 6). We found that the expression of the *TAF5* gene is significantly correlated with C20:3 and saturated and unsaturated FA contents.

## Discussion

### Significant differences in the mRNA expression profiles of the porcine GM and LD muscles

To the best of our knowledge, no report has been published so far comparing the transcriptomic profiles of two different muscles in pigs. Our differential expression analysis revealed that several loci encoding HOX homeodomain transcription factors (*HOXA9, HOXA10, HOXB6, HOXB7, ARX* and *TBX1*) are DE between the LD and GM muscles. These transcription factors have a well-known role in morphogenesis of developing embryos and, in adult vertebrates, they are involved in a plethora of functions related to cell division and muscle contractility [25]. Similarly, the comparison of the transcriptomic profiles of *gastrocnemius* and *quadriceps* muscles in mouse revealed that several genes involved in embryogenesis (i.e. *DKK3, HOXD8, HOXD9* and *TBX1*) are DE between these two muscles [26]. Moreover, Armstrong et al. [27] compared the expression profiles of nine lamb muscles by RNA-Seq and detected significant gene expression differences in almost all pairwise comparisons. Interestingly, the comparison of *semitendinosus* vs. *supraspinatus* mRNA levels showed that *HOXD8* and *HOXC10* genes are DE in these two muscles. Conceivably, HOX homeodomain transcription factors might play a critical role in the adult skeletal muscle of mice, sheep and pigs by controlling cell identity and differentiation of muscle fiber types.

The microarray analysis of gene expression also revealed that several genes related to the metabolism of glucose and lipids are DE between GM and LD muscles (Additional file 1 and Additional file 2). Consistently, Armstrong et al. [27] compared the transcriptomic profiles of the *semitendinosus*, *semimembranosus* and *longissimus lumborum* muscles vs. *suspraspinatus* and evidenced that carbohydrate metabolism pathways are enriched in the sets of DE genes. According to our results (Additional file 1), the fatty acid binding protein 3 (*FABP3*) mRNA, which encodes a molecule binding and transporting FA towards specific cell compartments [28], is overexpressed in the GM muscle. This finding is significant because it is known that fatty acid binding protein content in most cells is generally proportional to the rates of FA metabolism [28]. Moreover, a polymorphism of the porcine *FABP3* gene has been associated with IMF content and other lipid traits (reviewed in [3, 29]). Two other relevant genes that are downregulated in the GM muscle are the Nemo-like protein kinase (*NLK*), whose variation has been also associated with IMF content in pigs [30], and the nuclear receptor subfamily 2 group F member 1 (*NR2F1*) locus, an inhibitor of lipoprotein assembly in intestinal cells [31]. The protein kinase AMP-activated non-catalytic subunit ß_2_ (*PRKAB2*) gene, which was also downregulated in GM, encodes a subunit of the AMP-activated protein kinase, an enzyme that increases the rate of ß-oxidation of FA in the skeletal muscle [32]. With regard to the glucose metabolism, the solute carrier family 37 member 1 (*SLC37A1*) gene, which is overexpressed in GM, encodes a glucose-6-phosphate antiporter [33]. Glucose-6-phosphate dehydrogenase uses glucose-6-phosphate as a substrate to generate NADPH that can be used in FA biosynthesis [34]. These patterns of differential expression reflect differences in the lipid metabolism of LD and GM, a feature that could explain, at least in part, the higher IMF content of the GM (Additional file 7). Noteworthy, Morales et al. [35] observed differences in the activity of two lipogenic enzymes when comparing the GM and the *semimembranosus* muscles in a sample of Iberian and Landrace pigs.

### A genomic region on SSC6 is associated with intramuscular fat content in both GM and LD muscles

When backfat was used as a covariate to perform the GWAS for IMF content, we did not detect any significant association. In contrast, when we excluded this covariate from the statistical analysis, we found significant associations with IMF content in the GM (1 significant SNP) and LD (4 significant SNPs) muscles in a SSC6 region (146.5–147.7 Mb) which contains the leptin receptor gene (*LEPR*). Such association agrees well with previous studies showing that the polymorphism of the *LEPR* gene is associated with IMF content in Korean native × Yorkshire [36], Landrace × Iberian [37], Duroc × Iberian [38] and Duroc [12] pigs. The binding of leptin, a hormone secreted by the adipose tissue, to its receptor regulates satiety, body weight and the energy balance [39], so it could have potential effects on muscle fat deposition. Indeed, Ros-Freixedes and coworkers showed that the *LEPR* c.1987C > T SNP is associated with plasma leptin concentration, backfat thickness and IMF content in 853 Duroc pigs, suggesting the causal effects of this gene on fat deposition [12].

### A genomic region on SSC14 displays genome-wide significant associations with FA composition traits in both GM and LD muscles

The SSC14 region comprised between 110.9–114.5 Mb encompassed most of the associations observed in our study (Table 2, Fig. 2). In both muscles, marker effect sizes were negative for C18:0 and SFA and positive for C18:1 (n-7) and UFA, suggesting the existence of one causal mutation with opposed effects on both phenotypes or of at least two causal mutations with different effects on these phenotypes. This region had been identified in previous GWAS as associated with IMF composition. Yang et al. [7] carried out a GWAS for FA composition phenotypes and detected associations between SNPs on SSC14 (121 Mb, assembly version *Sscrofa10.2*) and LD C18:0 content in Sutai × (White Duroc × Erhualian) F_2_ pigs, whilst Zhang et al. [11] reported associations of this region with the C18:1(n-9)/C18:0 ratio in a related pig population. The same region was reported by Zhang et al. [10] and Sato et al. [4] as associated with LD C18:0 in Duroc × (Landrace × Yorkshire) crossbred pigs and in Duroc pigs, respectively. Consistently, Ros-Freixedes et al. [12] described that the SSC14 (110–114 Mb, assembly version *Sscrofa11.1*) region is associated with GM and LD C18:1, SFA, monounsaturated FA (MUFA) and the C18:1/C18:0 ratio, whereas Van Son et al. [5] indicated that this very same region displays associations with C16:0, C16:1, C18:0, C18:1(n-9), SFA and MUFA contents in subcutaneous fat. Altogether, these results support the existence of one or several genetic determinants on SSC14 (110.9–114.5 Mb) with multiple effects on muscle FA composition. Interestingly, the annotation of the QTL regions revealed that the SSC14 (110.9–114.5 Mb) region contains, in addition to 33 protein-coding genes, three microRNAs (ssc-miR-436, ssc-miR-146b and ssc-miR-1307) and 4 lincRNA genes (Additional file 4). Studies performed in humans indicate that miR-146b inhibits the proliferation of visceral preadipocytes and stimulates their differentiation [40], while the functions of the lincRNAs are mostly unknown.

In the analysis of candidate genes (Table 3), we have observed that SNPs mapping to the *KCNIP2* (rs320607389, SSC14: 112.86 Mb) and *BLOC1S2* (rs335981556, SSC14: 111.39 Mb) genes show significant associations with LD and GM C18:0 contents. However, the most obvious candidate locus to explain the associations detected in the SSC14 (110.9–114.5 Mb) region is the *SCD* gene, which encodes an enzyme involved in the desaturation of FA. Our results show the existence of significant associations between SNPs mapping to the *SCD* gene and GM and LD C18:0 and SFA (Table 3). Previously, Estany et al. [24] reported that the g.2228 T > C SNP (rs80912566) located in the 5’end of the porcine *SCD* gene is additively associated with the desaturation C18:1/C18:0 ratio in both muscle and subcutaneous fat in Duroc pigs, a result that would be consistent with ours. In a recent study, Fernández et al. [41] analysed the association of the *SCD* genotype and IMF composition in three (Iberian × Landrace) × Landrace, (Iberian × Duroc) × Duroc and (Iberian × Piétrain) × Piétrain backcrosses. These authors showed that the g.2228 T > C (rs80912566) SNP in the 5’end of the *SCD* gene is associated with LD C18:0 (*P*-value = 0.001) and SFA (*P*-value = 5.7 × 10^− 3^) contents but not with C18:1(n-9) content (*P*-value = 0.207) in the Duroc backcross. A similar outcome was obtained in the Piétrain backcross, highlighting the consistency of such results. In the GWAS carried out in the current work, the *ALGA0081091 S*NP, which shows the most significant associations with GM and LD C18:0 and C18:1(n-7), is located on SSC14:111,483,985 bp while the *SCD* g.2228 T > C SNP (rs80912566) maps to 14: 111,461,751 bp. In other words, there are ~ 22 kb between both markers. Sato et al. [4] and Van Son et al. [5] also reported that the SSC14 (120–124 Mb, assembly version *Sscrofa10.2,* 110–114 Mb in the *Sscrofa11.1* assembly) SNPs displaying the most significant associations with FA composition are located ~ 22 kb and 280,389 kb away from the *SCD* g.2228 T > C SNP, respectively, suggesting that *SCD* g.2228 T > C is linked to a yet to be found causal mutation with pleiotropic effects on FA traits.

The analysis of eQTL for 74 genes mapping to the SSC14 (110.9–114.5 Mb) QTL did not reveal any genetic determinant regulating *SCD* mRNA expression (Table 4). We also investigated differences in *SCD* mRNA expression amongst pigs with different g.2228 T > C SNP *SCD* genotypes (Additional file 3) and we did not find significant differences. Fernández et al. [41] compared the expression of the *SCD* mRNA in (Iberian × Duroc) × Duroc pigs with different haplotypes and found that individuals homozygous for the H_1_ *SCD* haplotype (which comprises the TT genotype) had a higher *SCD* mRNA expression in the LD muscle when compared with swine with other haplotypes, but differences were not statistically significant. Similar results were obtained by the same authors when analyzing *SCD* mRNA expression in (Iberian × Piétrain) × Piétrain pigs [41]. The g.2228 T > C SNP maps to the 5’UTR of the *SCD* gene, so it might have effects on the stability or the translatability of the transcript, but this needs to be confirmed with a functional assay.

By combining microarray and RNA-Seq data, we obtained evidence of eQTL regulating the expression of the *BLOC1S2* gene which maps to the SSC14 (110.9–114.5 Mb) QTL (Table 4). The *BLOC1S2* gene encodes a protein that forms part of the BLOC-1 complex, which is involved in the biogenesis and maturation of lysosomes and endosomes [42]. There are increasing evidences that lysosomes have a key role in the processing and sorting of exogenous and endogenous lipids [43]. Another gene of interest is *COX15*, which is involved in the assembly of cytochrome c oxidase, the terminal component of the mitochondrial respiratory chain [44]. The expression of these two genes did not significantly correlate with the phenotypic variation of C18:0, C18:1 (n-7), SFA or MUFA (Additional file 6). This could be due to the fact that the co-localizations detected by us do not have any functional meaning or, more likely, to the complex genomic architecture of these traits, which are determined by the action of multiple genes. On the other hand, there are many examples of genes whose expression levels do not directly correlate with protein levels and even less with protein activity levels. Therefore, although eQTL are an excellent starting point to detect sequence variability associated with gene activity, it is critical to assess also the potential consequences of regulatory polymorphisms with functional assays.

### Several genomic regions on pig chromosomes 2, 5, 9 and 18 show associations with fatty acid traits that are not consistently found inº both muscles

We have also identified several genomic regions i.e. SSC2 (10.6–11.7 Mb), SSC2 (21.9–22.3 Mb), SSC5 (76.2–77.1 Mb), SSC9 (10.4–13.2 Mb) and SSC18 (39.4–42.3 Mb) displaying genome-wide significant associations with LD C17:0, LD C16:1(n-9), GM C16:0, LD C14:0 and GM C20:3(n-3), respectively (Table 2 and Figs. 3 and 4). Amongst these, only the QTL for GM C16:0 on SSC5 and the QTL for GM C20:3(n-3) on SSC18 displayed effect sizes clearly different from zero (Table 2).

The association between SSC9 (10.4–13.2 Mb) and LD C14:0 has been consistently reported in the scientific literature. Sato et al. [4] and Zhang et al. [10] also described associations between SNPs comprised within this region and LD C14:0 content recorded in Duroc swine and Duroc × (Landrace × Yorkshire) crossbred pigs, respectively. Moreover, Zhang et al. [11] showed that this SSC9 region is associated with the C16:1(n-7)/C14:0 ratio in Duroc × (Landrace × Yorkshire) swine. On the other hand, genomic regions on SSC2 (10.6–11.7 Mb) and SSC5 (76.2–77.1 Mb) have been also associated with FA traits in Erhualian and in a meta-analysis comprising five pigs populations, but such associations differ from the ones reported by us with regard to the involved phenotype [11].

Our GWAS should have enough power to detect associations produced by SNPs with moderate to large effects, while SNPs with small effects would be undetectable (Additional file 3). In a similar study based on 331 pigs and a significance threshold of 1 × 10^− 6^, Zhang et al. [45] showed that the power to detect a QTL that explained 5% of the phenotypic variation was ≈23.6%, but it increased to 90% when the QTL explained 10% of the variance. In another study based on half-sib families of 60 individuals and a population size of 500 individuals, Teyssèdre et al. [46] showed that, when assuming h^2^ = 0.3–0.6, QTL explaining 4% of the variance of FA traits could be detected with a power of 80%. In the light of these results and ours, it is reasonable to infer that QTL with modest to large effects can be detected with a population of 300–400 pigs, while the majority of QTL with small effects would be missed.

The detection of different associations for traits recorded in the GM and LD muscles might be due to technical and biological factors. The size of the Duroc population analysed in the current work is modest and the precise quantification of QTL for minority FA can be challenging. Both factors might increase the rate of false negatives (type 2 error), thus generating spurious muscle-specific associations. We aimed to take a further look at this issue by genotyping a panel of selected SNPs mapping to QTL regions and investigating their associations with FA traits in the LD and GM muscles. The genotyping of the rs341329842 (SS5: 77.0 Mb) and rs333018168 (SSC5: 77.4 Mb) SNPs mapping to the *SLC38A1* and *SLC38A4* genes confirmed that they are associated with C16:0 in the GM but not in the LD muscle (Table 2). These two genes are involved in the transportation of glutamine [47], an amino acid that can be used as a substrate for lipogenesis [48], so it would be worth to further explore their role on the genetic determinism of FA composition. We also typed two SNPs, rs321243508 (SSC9:10.3 Mb) and rs328455999 (SSC9:11.3 Mb), which map to the *UVRAG* gene. This locus encodes a key regulator of autophagy [49], a biological process with an important impact on lipid storage [50]. The polymorphism of the *UVRAG* gene was associated with C14:0 in both muscles (Table 3), but the significance of such association was slightly higher for LD (q-value = 0.001) than for GM (q-value = 0.005). It is difficult to evaluate the relative impact of technical and biological factors on the lack of replication of several significant associations found either in LD or in GM. Carbonetto et al. [51] measured the weight of four hindlimb muscles in mice and, by using genotypes obtained with the high-density MegaMUGA SNP panel, they found that 18 and 15 genomic regions showed associations that were shared and non-shared across the four analysed muscles, respectively. In pigs, the existence of muscle-specific genomic associations with FA traits should be further investigated because such phenomenon may have practical implications in the framework of genomic selection schemes aimed to improve IMF composition traits.

## Conclusions

The comparison of the mRNA expression profiles of the LD and GM muscles has demonstrated the existence of significant differences in the mRNA expression of genes involved in the differentiation of muscle cells as well as in carbohydrate and lipid metabolism. This differential expression might be caused by differences in the body location, function and metabolism of the two porcine muscles under study. Performance of a GWAS has revealed an association between a region on SSC14 (110.9–114.5 Mb) and FA composition that happened to be highly significant in both GM and LD muscles. This region also contained *cis*-eQTL for genes with potential connections with lipid metabolism. In contrast, several of the associations reported in our study were not consistently found in both muscles, a finding that could be due to technical (e.g. lack of power) and/or biological factors.

## Methods

### Animal material and phenotype recording

Phenotypes were recorded in 350 barrows from a commercial Duroc line (Lipgen population) distributed in five half-sib families. After weaning, this pig population was transferred to the experimental test station at the Centre de Control Porcí of the Institut de Recerca i Tecnologia Agroalimentàries (IRTA). A detailed description of the experimental population and management conditions can be found in Gallardo et al. [52, 53]. Pigs were slaughtered at around 190 days of age (approximately 122 kg of live weight) following a commercial protocol in compliance with Spanish welfare regulations. Samples (200 g) of GM and LD muscles were taken immediately after slaughter to perform meat analyses at IRTA-Centre of Food Technology. A near infrared transmittance device (NIT, Infratec 1625, Tecator Hoganas, Sweden) was used to determine IMF content in the GM and LD muscles. The measurement of FA composition (C10 to C22 range) in the GM and LD muscles was achieved by gas chromatography of methyl esters [54]. A complete list of the IMF content and composition traits measured in the current experiment is shown in Additional file 7.

### Microarray-based analyses of muscle gene expression

For the current study, mRNA expression in the GM and LD muscles was analysed in 16 pigs chosen at random from the Lipgen population. This sample size should be sufficient to test if both muscles have similar or different transcriptomic profiles. *Gluteus medius* and LD muscle samples were collected from Duroc pigs at slaughter, snap frozen in liquid nitrogen and stored at − 80 °C. Both GM and LD mRNA expression profiles were characterized with the GeneChip Porcine Genome Array (Affymetrix, Inc., Santa Clara, CA). All details about RNA isolation, microarray hybridisation and quality control of expression data are provided in Cánovas et al. [55]. Microarray data describing GM and LD mRNA expression profiles were deposited in the Gene Expression Omnibus (GEO) public repository (GEO accession numbers: GSE19275 and GSE25708). The GeneChip Porcine Genomic arrays (ThermoFisher Scientific, Barcelona, Spain) were also used to measure mRNA expression in GM samples from 103 Duroc pigs (the 16 samples mentioned before plus 87 additional samples) with the aim of performing an eQTL scan. Data pre-processing, background correction, normalization and log-transformation of expression values between samples were carried out by computing a Robust Multi-array Average (RMA) as described by Irizarry et al. [56]. Differential expression between muscles (GM vs. LD) was assessed by following the limma-trend pipeline recommendations [57, 58], where the limma’s empirical Bayes procedure was modified to incorporate a mean-variance trend, modeling the relationship between variance and gene signal intensity. Fold-change (FC) values refer to mean expression levels in GM compared to LD.

Probes showing a |FC| > 1.5 and q-value < 0.05 were considered to be differentially expressed (DE) in the GM vs. LD comparison. Probes showing a significant DE were then translated to gene equivalents by using the Affymetrix porcine annotation data (chip porcine) assembled database [59] and the Biomart database available at Ensembl repositories (https://www.ensembl.org/biomart/martview/). Pathway enrichment analysis and gene ontology annotations were performed upon the more stringent filtering of DE probes (|FC| > 1.5 and q-value < 0.05) using Panther Gene List Classification System (http://www.pantherdb.org).

### Genotyping

Whole-genome genotyping of the 350 Duroc pigs was performed using the Porcine SNP60 BeadChip (Illumina, San Diego, CA) which contains probes for 62,163 single nucleotide polymorphisms (SNPs). Filtering analyses based on the quality of the genotyping results were performed with the GenomeStudio software (Illumina). By using PLINK v. 1.07 [60], we filtered SNPs with minor allele frequencies below 5%, rates of missing genotypes above 10% or showing highly significant departures from the Hardy-Weinberg expectation (threshold set at a *P*-value of 0.001). The SNPs that did not map to the porcine reference genome (*Sscrofa11.1* assembly) and those located in sexual chromosomes were also excluded from further analyses. After these filtering steps, a subset of 32,784 SNPs were used as markers in the genome-wide association study (GWAS).

Additionally, 12 SNPs mapping to the solute carrier family 38 member 1 (*SLC38A1,* rs341329842) and member 4 (*SLC38A4,* rs333018168), UV radiation resistance associated (*UVRAG,* and rs328455999), biogenesis of lysosomal organelles complex 1 subunit 2 (*BLOC1S2*, rs335981556), potassium voltage-gated channel interacting protein 2 (*KCNIP2,* rs320607389) and stearoyl-CoA desaturase (*SCD,* rs335981556, rs698797651, rs323081995, rs80912566, rs342182479, rs45434498 and rs713641545) genes were genotyped in the Veterinary Service of Molecular Genetics (http://svgm.es/ca/Home) of the Universitat Autònoma de Barcelona by using a QuantStudio 12 K Flex real-time PCR instrument (Thermo Fisher Scientific). Primers are listed in Additional file 8. These SNPs were identified by taking into account the whole-genome sequencing of the five parental boars (our unpublished data) as well as by the RNA-Sequencing of 52 pigs selected from the population of 350 offspring individuals [23]. Polymorphisms were selected according to their co-localization with the QTL identified in the current work as well as by their potential effect on gene function (SNPs at splice sites or causing amino acid substitutions were prioritized). We also prioritized SNPs that were segregating in our resource Duroc population.

### Genome-wide and gene-centric association analyses with intramuscular fat phenotypes

Statistical methods employed in the current work have been previously reported by González-Prendes et al. [61]. In this way, mixed-model association analyses were carried out with the Genome-wide Efficient Mixed-Model Association (GEMMA) software, developed by Zhou and Stephens [62]. This method corrects population structure by considering the relatedness matrix, which is built by taking into account all genome-wide SNPs as a random effect. We used the following statistical model to estimate each SNP effect on IMF content and composition traits:

1 $$ \boldsymbol{y}=\mathbf{W}\boldsymbol{\upalpha } +\mathbf{x}\ \updelta +\mathbf{u}+\boldsymbol{\upvarepsilon} $$

where ***y*** is the vector of phenotypic values for all individuals; **W** is a matrix including a column of 1 s, the incidence of fixed effects (batch of fattening, with 4 categories) plus a covariate that depends on the trait: (1) IMF content in GM (for FA traits measured in the GM muscle), (2) IMF content in LD (for FA traits measured in the LD muscle), (3) backfat thickness (for IMF content measured in GM and LD); **α** is a vector of the corresponding fixed effects that includes the intercept, the batch effects and the regression coefficient on the covariate; **x** is a vector of marker genotypes in each individual; **δ** is the effect size of the marker (allele substitution effect); **u** is a vector of random individual genetic effects with a n-dimensional multivariate normal distribution **u** ∼ N (**0**, λ *τ*^− 1^ **K**), being *τ*^− 1^ the variance of the residual error, **λ** is the ratio between the two variance components and **K** a known relatedness matrix derived from SNP genotypes; and **ε** is the vector of errors. The statistical relevance of the systematic environmental sources of variation and the covariates were previously corroborated by Gallardo et al. [52] and Casellas et al. [63]. In this study, QTL were defined as those genomic regions containing at least 2 SNPs significantly associated with a given IMF content or composition trait. Correction for multiple testing was implemented with a false discovery rate (FDR) approach [64]. The gene-centric association analysis for the *SLC38A1, SLC38A4, UVRAG, BLOC1S2, KCNIP2* and *SCD* loci was performed with GEMMA by using the same methods reported above. The power of the GWAS to detect associations in a population of 350 Duroc pigs was evaluated with the Genetic Power Calculator software [22]. We assumed equal allele frequencies between an unobserved causal variant and an observed genotype in our panel of SNPs under two different scenarios of linkage disequilibrium (r^2^ = 0.6 and r^2^ = 0.8). The type I error rate (α) was fixed to α = 0.00005 (equivalent to a q-value = 0.05 in our associations test). The effect size of the SNP (β) expressed in standard deviations (we assumed a normal distribution) and a minor allele frequency (MAF) equal to 0.35 were taken into account to calculate the variance explained by the causal variant in the population (q^2^ = β^2^*MAF).

### Performance of genome-wide and gene-centric association analyses with gene expression phenotypes

We performed a genome scan to identify potential *cis*-eQTL regulating the expression of 74 genes mapping to QTL determining IMF traits recorded in the GM muscle by using a previously reported methodology [61]. Gene expression phenotypes were determined with microarrays, as reported above. Official gene names and positions of each probe included in the GeneChip Porcine Genomic array (ThermoFisher, Barcelona, Spain) were identified in the BioMart database [65]. The statistical model assumed in the GEMMA analysis was the same reported in the previous section. However, the vector of fixed effects **α** and its corresponding incidence matrix **W** included not only the batch of fattening (with 4 categories) but also a “laboratory” fixed factor (two levels) because microarray data were generated in two different laboratories. Correction for multiple testing was implemented with the FDR approach mentioned above [64]. The threshold of significance in the analysis of *cis-*eQTL was established in accordance with previously reported criteria [61]. We considered that two QTL and eQTL co-localize when there is at least an overlap of 1 base pair between the genomic regions containing them. The set of *cis*-eQTL identified with the methods reported above were independently confirmed by using RNA-Seq data from 52 of the 103 pigs employed in the current experiment. The methods involved in the RNA-Seq experiment have been reported by Cardoso et al. [23].

Additionally, we evaluated the effects of g.2228 T > C SNP *SCD* genotypes over the *SCD* mRNA expression (measured with microarrays and RNA-Seq) with a linear model considering the batch and lab effects. The significance of mean differences between TT, TC and CC genotypes were assessed with a Student’s t-test [66].

## Additional files

Additional file 1: **Table S1.** List of genes differentially expressed between the *gluteus medius* and *longissimus dorsi* muscles (|FC| > 1.5; q-value< 0.05). (XLSX 18 kb)

Additional file 2: **Table S2.** Pathway Enrichment for genes differentially expressed between the *gluteus medius* and *longissimus dorsi* muscles (|FC| > 1.5; q-value < 0.05). (XLSX 12 kb)

Additional file 3: **Figure S1.** Genetic Power Calculator plots showing the power of the GWAS analysis (x-axis) for a population of 350 Duroc pigs. The effect size (from 0 to 0.35) is plotted in the y-axis. We have assumed an allele frequency of 0.35 and two potential r^2^ values (0.6 and 0.8) between the unknown causal mutation and the marker SNP. **Figure S2.** Boxplots depicting the mRNA expression levels of 5 cis-eQTL regulated genes measured with RNA-Seq and microarrays in the *gluteus medius* muscle of 52 and 103 Duroc pigs, respectively. Means were compared with a Student’s t- test: *P*-value > 0.05 (ns); *P*-value ≤0.05 (*); *P*-value ≤0.01 (**); *P*-value ≤0.001 (***) and *P*-value ≤0.0001 (****). **Figure S3.** Least square means of the stearoyl-CoA desaturase (*SCD*) mRNA expression levels measured with microarrays (TT, *N* = 4; TC, *N* = 20 and CC, *N* = 18) and RNA-Seq (TT, N = 4; TC, N = 20 and CC, N = 18) in the *gluteus medius* muscle of Duroc pigs with known genotypes for a polymorphism located in the 5’end of the porcine *SCD* gene (g.2228 T > C). Means were compared with a Student’s t- test. Although in both analyses CT pigs show a higher *SCD* mRNA expression than the CC and TT ones, differences are non-significant (ns). (DOCX 421 kb)

Additional file 4: **Table S3.** List of microRNA and long intergenic non-coding RNA genes mapping to the SSC14 QTL identified in the current work. (XLSX 10 kb)

Additional file 5: **Table S4.** Genes located within QTL regions retrieved from the list of genes showing differential expression between the *gluteus medius* and *longissimus dorsi* muscles at different thresholds of fold-change (FC) and statistical significance. (XLSX 12 kb)

Additional file 6: **Table S5.** Correlations between the expression of genes regulated by *cis*-eQTL and the variation of phenotypes determined by QTL co-localizing with the corresponding *cis*-eQTL (the statistical significance of the correlation is expressed as a *P*-value between parentheses). (DOCX 19 kb)

Additional file 7: **Table S6.** Means and standard deviations (SD) of intramuscular fat and composition traits recorded in two porcine muscles (*N* = 350). (DOCX 14 kb)

Additional file 8: **Table S7.** Primer sequences used in the amplification of SNPs mapping to candidate genes located within QTL regions associated with fatty acid composition traits. (XLSX 11 kb)

## Abbreviations

|FC|: Absolute value of the fold-change
bp: Base pair
DE: Differentially expressed
e.g.: For example
eQTL: Expression QTL
FA: Fatty acids
GEMMA: Genome-wide Efficient Mixed-Model Association
GEO: Gene Expression Omnibus
GM: *Gluteus medius*
GWAS: Genome-wide association analysis
i.e.: In other words
IMF: Intramuscular fat
Kb: Kilobase pairs
LD: *Longissimus dorsi*
lincRNA: Long intergenic noncoding RNA
Mb: Megabase pairs
mRNA: Messenger RNA
QTL: Quantitative trait loci
r_P_: Phenotypic correlation
SNP: Single nucleotide polymorphism
SSC: Porcine chromosome
δ_GM_: Marker effect size for traits measured in the *gluteus medius* muscle
δ_LD_: Marker effect size for traits measured in the *longissimus dorsi* muscle
