# Supplementary material for: Comparing the mRNA expression profile and the genetic determinism of intramuscular fat traits in the porcine gluteus medius and longissimus dorsi muscles
Source: BMC Genomics. 2019 Mar 4;20:170. doi: 10.1186/s12864-019-5557-9 (PMC6399881; doi:10.1186/s12864-019-5557-9)

Figure S1. Genetic Power Calculator plots showing the power of the GWAS analysis (y-axis) for a population of 350 Duroc pigs. The effect size (from 0 to 0.35) is plotted in the y-axis. We have assumed an allele frequency of 0.35 and two potential r^2^ values (0.6 and 0.8) between the unknown causal mutation and the marker SNP.


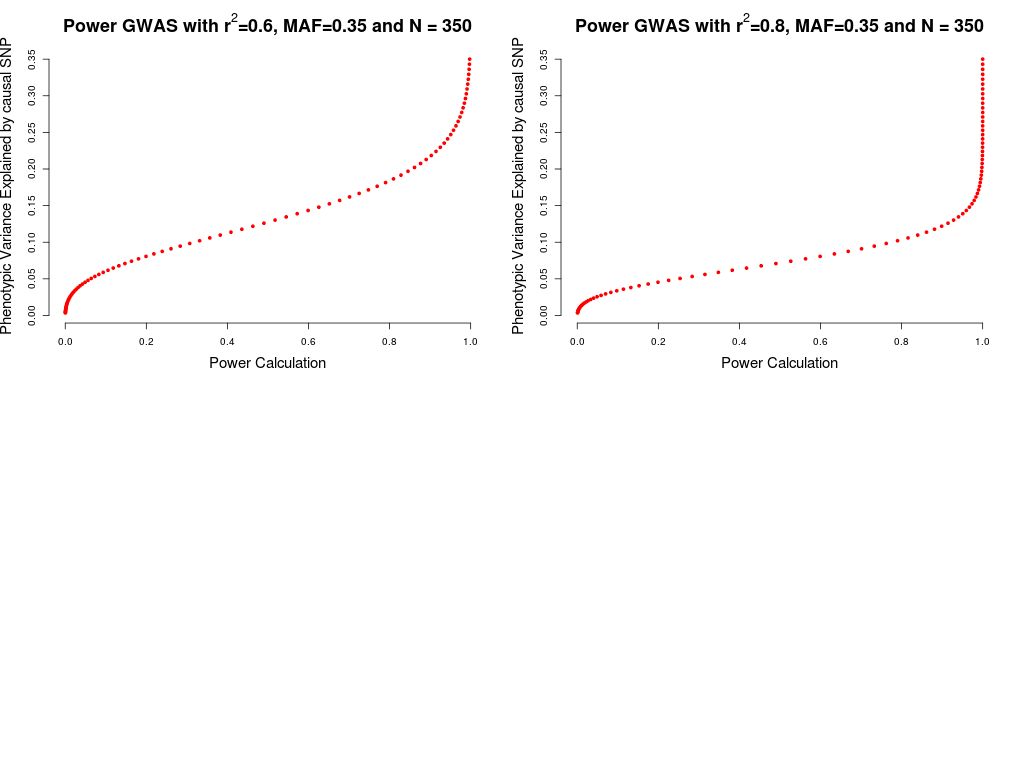


Figure S2. Boxplots depicting the mRNA expression levels of cis-eQTL regulated genes measured with RNA-Seq and microarrays in the *gluteus medius* muscle of 52 and 103 Duroc pigs, respectively. Means were compared with a Student’s t- test: *P*-value > 0.05 (ns); *P*-value ≤ 0.05 (*); *P*-value ≤ 0.01 (**); *P*-value ≤ 0.001 (***) and *P*-value ≤ 0.0001 (****).


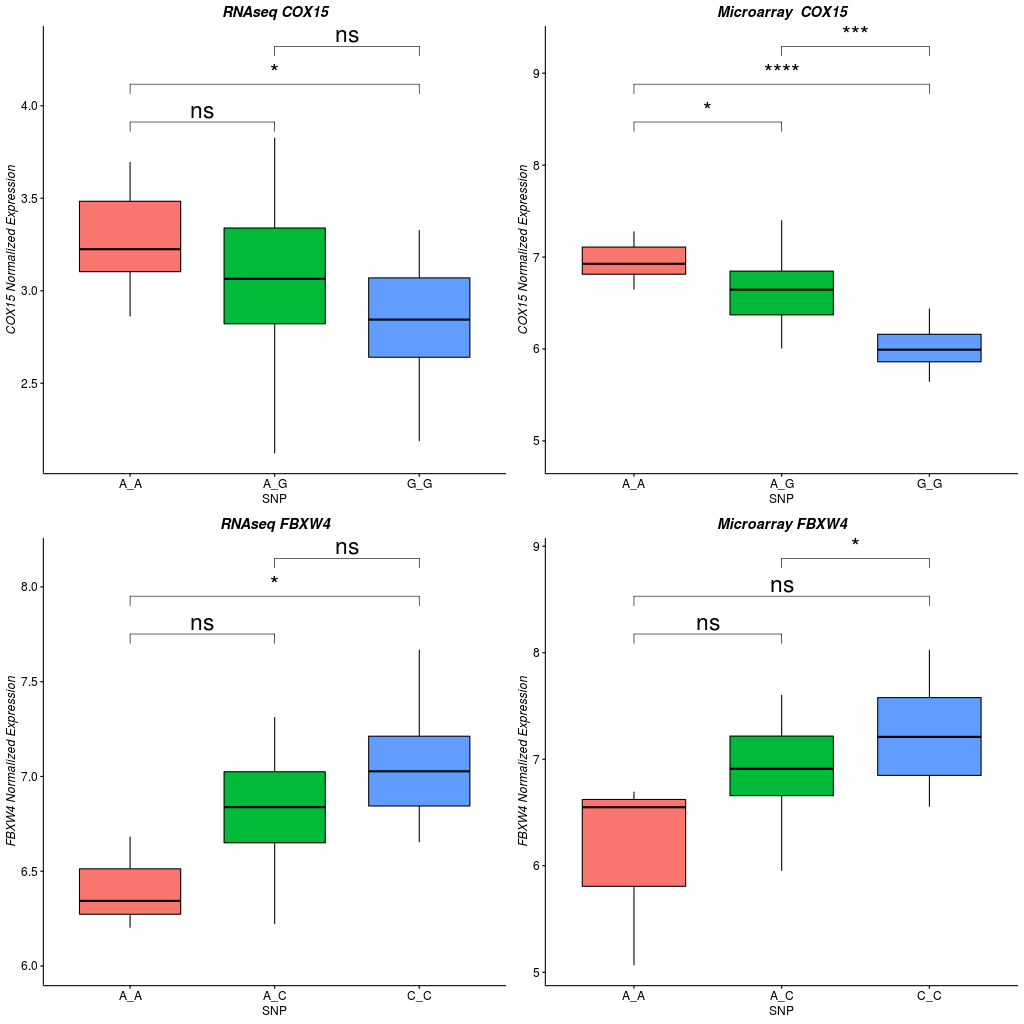

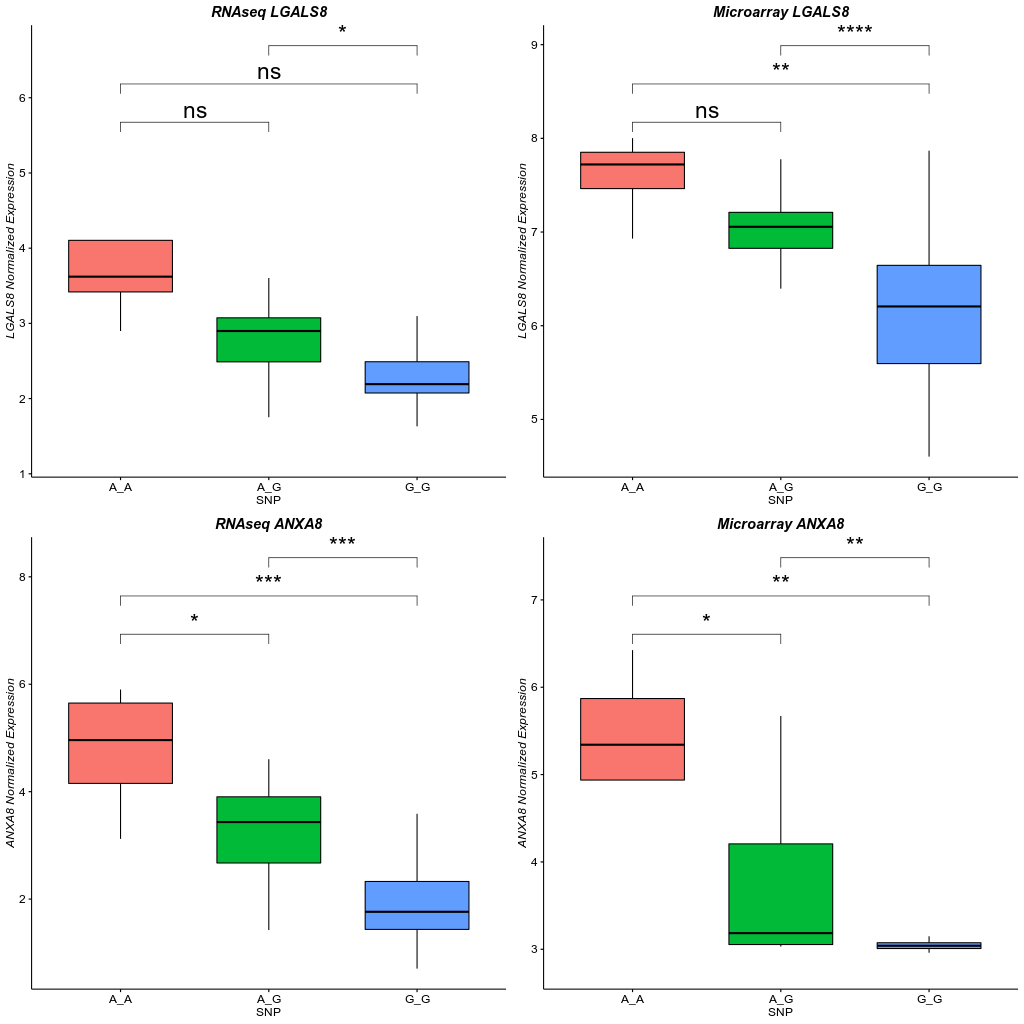


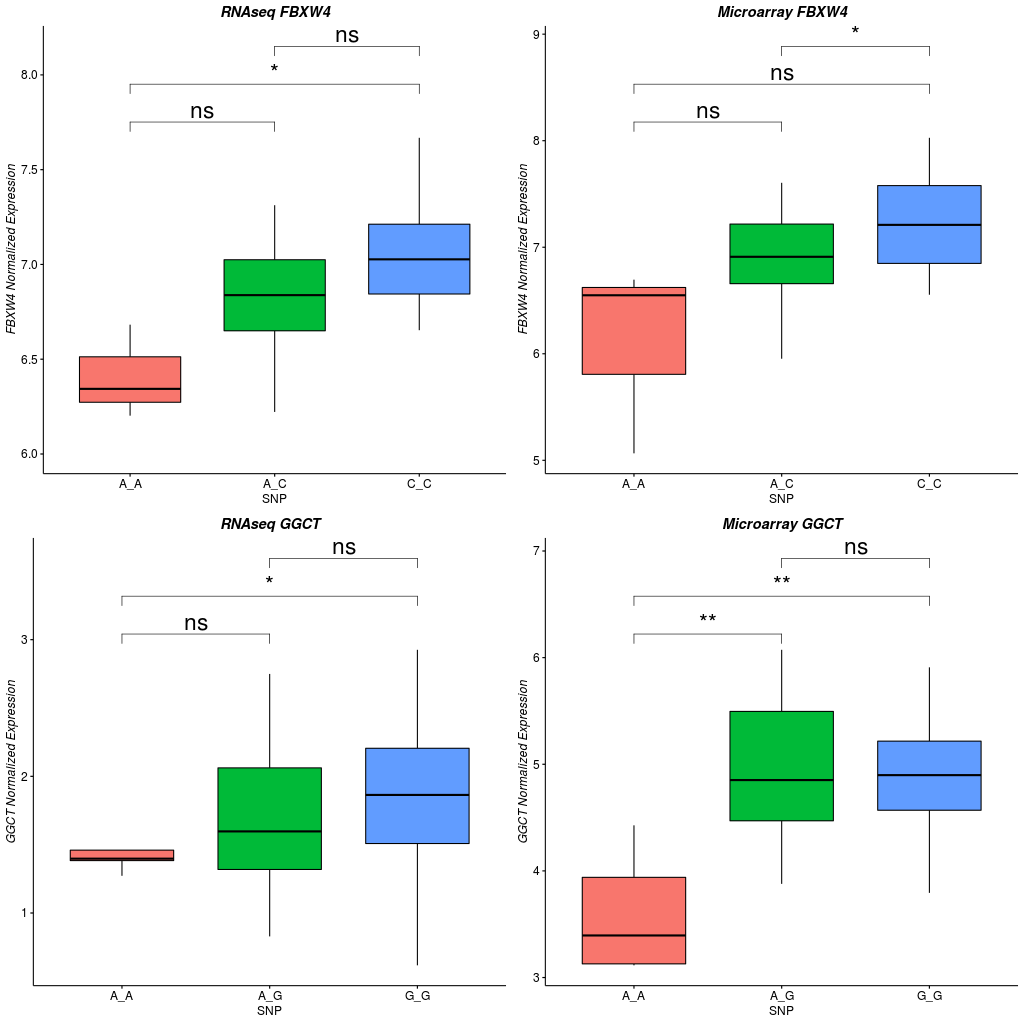


Figure S3. Least square means of the stearoyl-CoA desaturase (*SCD*) mRNA expression levels measured with microarrays (TT, N=4; TC, N=20 and CC, N=18) and RNA-Seq (TT, N=4; TC, N=20 and CC, N=18) in the *gluteus medius* muscle of Duroc pigs with known genotypes for a polymorphism located in the 5’end of the porcine *SCD* gene (g.2228T > C). Means were compared with a Student’s t- test. Although in both analyses CT pigs show a higher *SCD* mRNA expression than the CC and TT ones, differences are non-significant (ns).


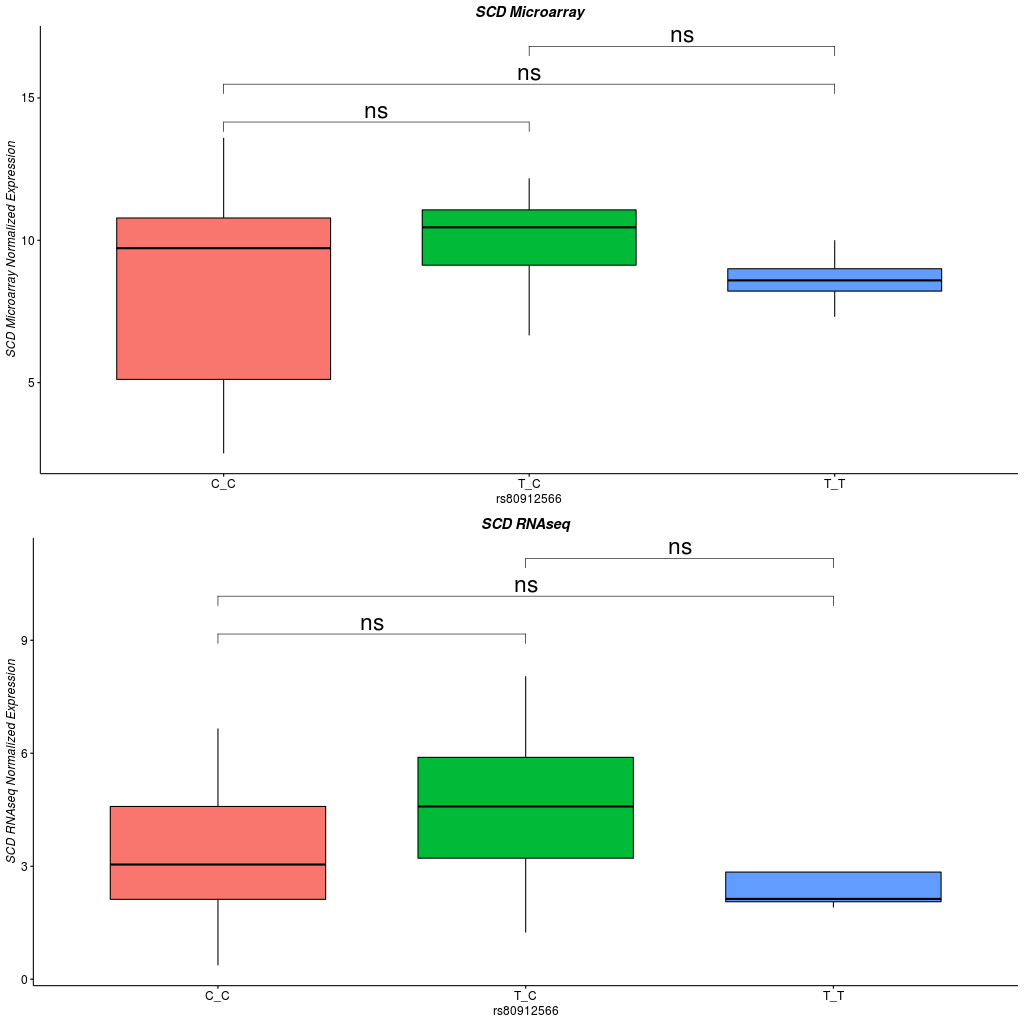

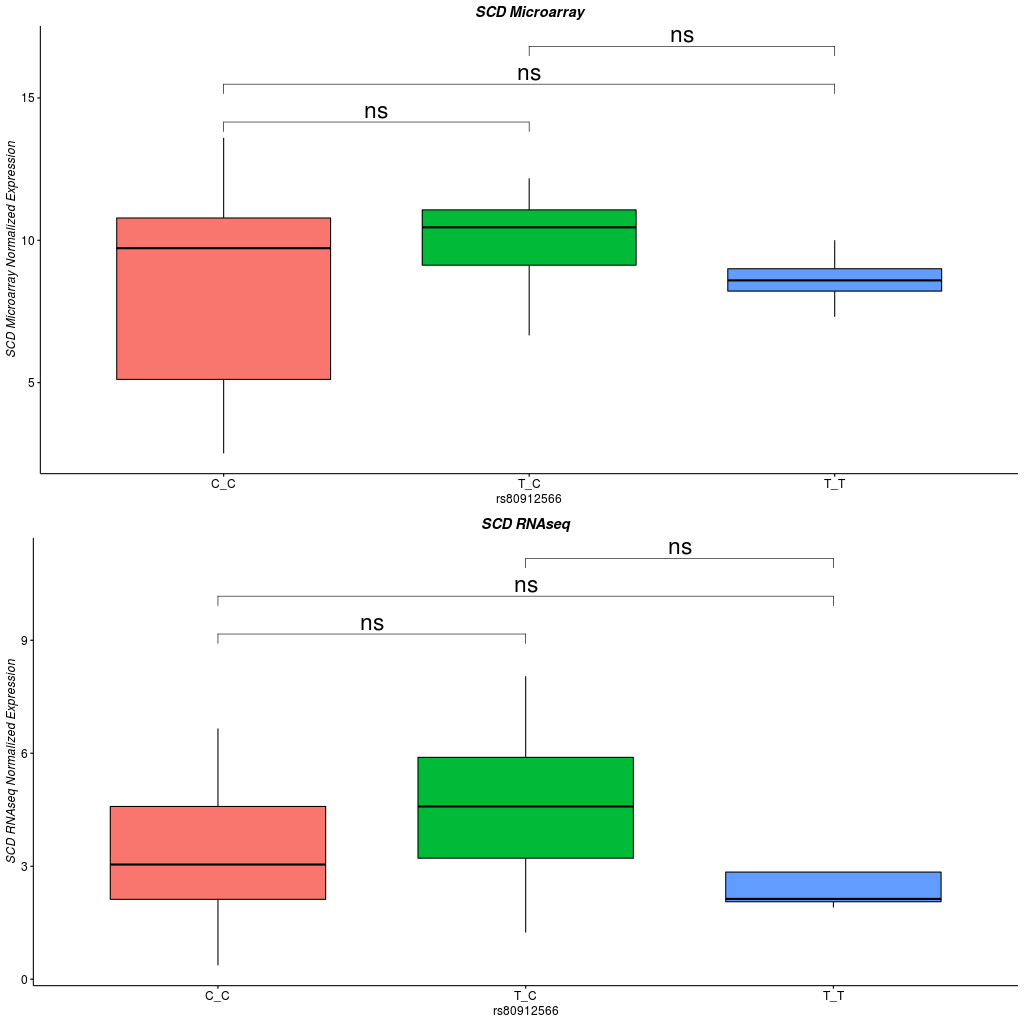

Supplement: Supplementary file 3 — Figure S1. Genetic Power Calculator plots showing the power of the GWAS analysis (x-axis) for a population of 350 Duroc pigs. The effect size (from 0 to 0.35) is plotted in the y-axis. We have assumed an allele frequency of 0.35 and two potential r2 values (0.6 and 0.8) between the unknown causal mutation and the marker SNP. Figure S2. Boxplots depicting the mRNA expression levels of 5 cis-eQTL regulated genes measured with RNA-Seq and microarrays in the gluteus medius muscle of 52 and 103 Duroc pigs, respectively. Means were compared with a Student’s t- test: P-value > 0.05 (ns); P-value ≤0.05 (*); P-value ≤0.01 (**); P-value ≤0.001 (***) and P-value ≤0.0001 (****). Figure S3. Least square means of the stearoyl-CoA desaturase (SCD) mRNA expression levels measured with microarrays (TT, N = 4; TC, N = 20 and CC, N = 18) and RNA-Seq (TT, N = 4; TC, N = 20 and CC, N = 18) in the gluteus medius muscle of Duroc pigs with known genotypes for a polymorphism located in the 5’end of the porcine SCD gene (g.2228 T > C). Means were compared with a Student’s t- test. Although in both analyses CT pigs show a higher SCD mRNA expression than the CC and TT ones, differences are non-significant (ns). (DOCX 421 kb) [file 12864_2019_5557_MOESM3_ESM.docx]
