# Supplementary material for: Comparing the mRNA expression profile and the genetic determinism of intramuscular fat traits in the porcine gluteus medius and longissimus dorsi muscles
Source: BMC Genomics. 2019 Mar 4;20:170. doi: 10.1186/s12864-019-5557-9 (PMC6399881; doi:10.1186/s12864-019-5557-9)
Supplement: Supplementary file 6 — Table S5. Correlations between the expression of genes regulated by cis-eQTL and the variation of phenotypes determined by QTL co-localizing with the corresponding cis-eQTL (the statistical significance of the correlation is expressed as a P-value between parentheses). (DOCX 19 kb) [file 12864_2019_5557_MOESM6_ESM.docx]

Table S5. Correlations between the expression of genes regulated by *cis*-eQTL and the variation of phenotypes determined by QTL co-localizing with the corresponding *cis*-eQTL (the statistical significance of the correlation is expressed as a *P*-value between parentheses).

| **Ensembl ID** | **Gene Symbol** | **C18:0** | **C18:1(n7)** | **C20:3** | **SFA** | **UFA** |
| --- | --- | --- | --- | --- | --- | --- |
| ***ENSSSCG00000010555*** | *HIF1AN* | -0.0679 (0.4956) | -0.0936 (0.3469) | - | -0.0643 (0.5185) | 0.0643 (0.5185) |
| ***ENSSSCG00000010370*** | *ANXA8* | -0.1241 (0.2116) |  | - | - | - |
| ***ENSSSCG00000010551*** | *BLOC1S2* | -0.1080 (0.2777) | 0.0879 (0.3773) | - | 0.1073 (0.2805) | -0.1073 (0.2805) |
| ***ENSSSCG00000010146*** | *LGALS8* | -0.0735 (0.4606) |  | - | - | - |
| ***ENSSSCG00000010541*** | *COX15* | 0.0489 (0.6235) | 0.0176 (0.8597) | - | 0.1366 (0.1690) | -0.1366 (0.1690) |
| ***ENSSSCG00000016679*** | *GGCT* | - | - | 0.0974 (0.3278) | - | - |
| ***ENSSSCG00000016685*** | *WIPF3* | - | - | 0.1208 (0.2243) | - | - |
| ***ENSSSCG00000010571*** | *ARMH3* | -0.0985 (0.3223) | 0.1440 (0.1468) | - | 0.0673 (0.4993) | -0.0673 (0.4993) |
| ***ENSSSCG00000010566*** | *FBXW4* | 0.0117 (0.9068) | 0.0780 (0.4335) | - | 0.1074 (0.2804) | -0.1074 (0.2804) |
| ***ENSSSCG00000037575*** | *FKBP14* | - | - | 0.1190 (0.2311) | - | - |
| ***ENSSSCG00000040337*** | *AK4* | - | - | - | - | - |
| ***ENSSSCG00000033190*** | *AQP1* | - | - | **-0.1830 (0.0643)** | - | - |
| ***ENSSSCG00000010570*** | *KCNIP2* | 0.0177 (0.8590) | 0.0235 (0.8139) | - | -0.1234 (0.2143) | 0.1234 (0.2143) |
| ***ENSSSCG00000010597*** | *TAF5* | -0.0042 (0.9661) | -0.1036 (0.2978) | **0.2672 (0.0064)-** | **0.2691 (0.0060)** | **-0.2691 (0.0060)** |
| ***ENSSSCG00000010151*** | *LYST* | 0.0521 (0.6012) | - | - |  |  |
