# Supplementary material for: Comparing the mRNA expression profile and the genetic determinism of intramuscular fat traits in the porcine gluteus medius and longissimus dorsi muscles
Source: BMC Genomics. 2019 Mar 4;20:170. doi: 10.1186/s12864-019-5557-9 (PMC6399881; doi:10.1186/s12864-019-5557-9)
Supplement: Supplementary file 7 — Table S6. Means and standard deviations (SD) of intramuscular fat and composition traits recorded in two porcine muscles (N = 350). (DOCX 14 kb) [file 12864_2019_5557_MOESM7_ESM.docx]

Table S6. Means and standard deviations (SD) of intramuscular fat and composition traits recorded in two porcine muscles (N = 350).

| **Phenotype (%)** | **Symbol** | | ***longissimus dorsi* muscle** | ***gluteus medius* muscle** | |
| --- | --- | --- | --- | --- | --- |
|  |  |  | **Mean ± SD** | **Mean ± SD** | |
| **Intramuscular fat** | **IMF** | | **3.91±1.53** | **5.2±2.05** | |
| **Saturated FA** | **SFA** | | **37.11 ± 2.41** | **36.47 ± 2.07** | |
| Capric | C10:0 | | 0.10 ± 0.07 | 0.11 ± 0.06 | |
| [Lauric](https://en.wikipedia.org/wiki/Lauric_acid) | C12:0 | | 0.09 ± 0.04 | 0.09 ± 0.04 | |
| Myristic | C14:0 | | 1.37 ± 0.27 | 1.39 ± 0.23 | |
| Palmitic | C16:0 | | 23.47 ± 1.64 | 23.23 ± 1.42 | |
| Margaric | C17:0 | | 0.21 ± 0.08 | 0.27 ± 0.16 | |
| Stearic | C18:0 | | 11.71 ± 1.22 | 11.21 ± 1.13 | |
| Arachidic | C20:0 | 0.18 ± 0.07 | | | 0.17 ± 0.12 |
| **Unsaturated FA** | **UFA** | | **62.89 ± 2.41** | **63.53 ± 2.07** | |
| **Monounsaturated FA** | **MUFA** | | **43.29 ± 5.65** | **43.19 ± 4.9** | |
| Palmitoleic | C16:1 (n-7) | | 2.98 ± 0.59 | 2.82 ± 0.49 | |
| Palmitelaidic | C16:1 (n-9) | | 0.24 ± 0.04 | 0.28 ± 0.05 | |
| Heptadecenoic | C17:1 | | 0.17 ± 0.06 | 0.21 ± 0.06 | |
| Octadecenoic | C18:1 (n-7) | | 4.29± 0.34 | 4.06±0.31 | |
| Oleic | C18:1 (n-9) | | 34.96 ± 5.16 | 35.13 ± 4.48 | |
| Gondoic | C20:1 | | 0.66 ± 0.16 | 0.68 ± 0.14 | |
| **Polyunsaturated FA** | **PUFA** | | **19.6 ± 7.35** | **20.35 ± 6.04** | |
| Linoleic | C18:2 | | 14.12 ± 5.09 | 14.93 ± 4.1 | |
| α-Linolenic | C18:3 (n-3) | | 0.48 ± 0.09 | 0.62 ± 0.1 | |
| Eicosadienoic | C20:2 (n-6) | | 0.41 ± 0.1 | 0.53 ± 0.14 | |
| Eicosatrienoic | C20:3 (n-3) | | 0.14 ± 0.09 | 0.18 ± 0.17 | |
| Arachidonic | C20:4 | | 3.52 ± 1.8 | 3.18 ± 1.55 | |
| Eicosapentaenoic | C20:5 | | 0.16 ± 0.12 | 0.18 ± 0.14 | |
| Docosahexaenoic | C22:6 | | 0.12 ± 0.1 | 0.12 ± 0.12 | |
| **Omega-3 FA** | **FA n-3** | | **0.91 ± 0.29** | **1.10 ± 0.34** | |
| **Omega-6 FA** | **FA n-6** | | **18.69 ± 7.12** | **19.25 ± 5.81** | |
| **Omega-6 to -3 ratio** | **n-6/n-3** | | **20.43 ± 4.71** | **17.82 ± 3.86** | |
